# Development and Initial Validation of the Multidimensional Psychosocial Work Environment Scale for Employed Persons (MPWES)

**DOI:** 10.3390/ijerph23070854

**Published:** 2026-06-30

**Authors:** Evija Nagle, Iluta Skrūzkalne, Maksims Zolovs, Olga Rajevska, Otto Andersen, Andrejs Ivanovs, Ieva Reine

**Affiliations:** 1 Statistics Unit, Rīga Stradiņš University, Dzirciema iela 16, LV-1007 Riga, Latvia; 2 Institute of Life Sciences and Technology, Daugavpils University, Vienības iela 13, LV-5401 Daugavpils, Latvia; 3 School of Business and Law, University of Agder, Gimlemoen 25, 4630 Kristiansand, Norway; 4 Department of Public Health and Caring Sciences, Uppsala University, Husargatan 3, SE-751 22 Uppsala, Sweden

**Keywords:** psychosocial work environment, employee well-being, scale development, psychometric validation, confirmatory factor analysis, occupational health

## Abstract

**Highlights:**

**Public health relevance—How does this work relate to a public health issue?**

- Psychosocial work environment factors, including job demands, workplace risks, social support, and autonomy, are closely associated with employee mental health, subjective well-being, and workforce sustainability. However, existing instruments often assess these domains separately. This study addresses an important public health need by developing and initially validating the Multidimensional Psychosocial Work Environment Scale for Employed Persons (MPWES), an integrated instrument designed to assess psychosocial work environment factors and employee subjective well-being simultaneously across occupational sectors.

**Public health significance—Why is this work of significance to public health?**

- Poor psychosocial working conditions are associated with burnout, psychological distress, reduced work ability, absenteeism, and long-term health consequences. Early identification of psychosocial risks and workplace resources is therefore essential for prevention and workforce health promotion. The MPWES provides a multidimensional and psychometrically evaluated framework that may support more comprehensive psychosocial work environment assessment and contribute to improved occupational health monitoring and employee well-being evaluation.

**Public health implications—What are the key implications or messages for practitioners, policy makers and/or researchers in public health?**

- The MPWES may support occupational health specialists, organizations, and public health researchers in identifying psychosocial workplace risks and resources associated with employee well-being. The instrument may contribute to workplace mental health promotion, psychosocial risk management, and evidence-based organizational prevention strategies. In addition, the scale may support the development of sustainable workplace policies and ESG-related social monitoring practices. Further longitudinal and cross-cultural validation studies are recommended before broader implementation.

**Abstract:**

Background: Psychosocial well-being at work is a multidimensional construct associated with employee health, organizational functioning, sustainable workforce development, and population mental health. However, few theoretically integrated instruments simultaneously assess work-related resources, job demands, psychosocial risks, and employee subjective well-being. This study aimed to develop and conduct the initial validation of the Multidimensional Psychosocial Work Environment Scale for Employed Persons (MPWES), grounded in the OECD well-being framework, the WHO-5 conceptual approach, and the Job Demands–Resources model. Methods: Scale development involved theory-driven identification of psychosocial dimensions, item generation, content and face validity assessment, and stepwise psychometric evaluation. Content validity was assessed using the Content Validity Index, and face validity using the Face Validity Index. The hypothesized structure was examined using confirmatory factor analysis. Internal consistency was evaluated using Cronbach’s alpha and McDonald’s omega, while convergent and discriminant validity were assessed using Average Variance Extracted, Composite Reliability, and inter-factor correlations. Results: The proposed measurement model comprised ten dimensions: Subjective Well-Being, Inclusion, Social Support, Workplace Harassment, Work Intensity, Work-related Psychosomatic Strain, Professional Development, Health Risks, Financial Safety, and Autonomy. CFA results provided preliminary and partial support for the proposed ten-factor structure, with borderline-to-acceptable absolute fit indices but limited-to-moderate incremental fit indices. Most dimensions demonstrated acceptable internal consistency; however, convergent and discriminant validity findings should be interpreted cautiously, particularly for dimensions with few items, Financial Safety, and the high correlation between Inclusion and Social Support. Conclusions: The findings provide preliminary empirical support for the MPWES as an initial integrated assessment framework. Further longitudinal, cross-cultural, and independent validation is required.

## 1. Background

The psychosocial work environment has become an increasingly important area of research in occupational health and public health due to its substantial influence on employees’ mental health, subjective well-being, work ability, and workforce sustainability [1,2,3]. Employees across different occupational sectors are frequently exposed to high work intensity, emotional strain, limited autonomy, inadequate social support, and various psychosocial risks that may negatively affect individual psychological functioning and organizational effectiveness [1,4]. Previous studies have demonstrated that adverse psychosocial working conditions are associated with burnout, presenteeism, psychological distress, lower work engagement, increased sickness absence, and higher turnover intentions [3,4]. Conversely, positive job resources, such as social support, autonomy, and opportunities for professional development, have been identified as important protective factors that promote employees’ subjective well-being and occupational resilience [1,5].

In occupational health research, subjective well-being (SWB) is generally conceptualized as an individual psychological state encompassing emotional well-being, positive functioning, and perceived psychological balance within the work context [6,7]. At the same time, psychosocial work environment factors, including job demands, job resources, and psychosocial risks, are conceptualized as external workplace characteristics that may be associated with employees’ subjective well-being [1,8]. Thus, subjective well-being and psychosocial work environment factors are conceptually related but theoretically distinct constructs. This distinction is central to the conceptual logic of the MPWES: subjective well-being is treated as an outcome-related psychological dimension, whereas the remaining dimensions represent workplace-level psychosocial conditions that may support, challenge, or undermine employee well-being.

Previous studies conducted in Latvia have demonstrated that healthcare professionals’ subjective well-being is substantially influenced by psychosocial work environment factors, including work intensity, emotional strain, autonomy, and organizational ethical climate [9,10]. Furthermore, significant sectoral differences in psychosocial risks and job resources have been identified across occupational settings [11]. These findings highlight the need for an integrated approach that enables the simultaneous assessment of psychosocial risks, job resources, and subjective well-being dimensions within a unified occupational assessment framework.

Therefore, the present study introduces the Multidimensional Psychosocial Work Environment Scale for Employed Persons (MPWES). The instrument was developed based on the Job Demands–Resources (JD-R) model [1], the OECD job quality framework [8], and conceptual principles of subjective well-being [6,7]. MPWES was designed as an integrated psychosocial work environment assessment instrument that incorporates dimensions related to job demands, job resources, psychosocial risks, and subjective well-being while conceptually distinguishing workplace factors from subjective well-being as a psychological outcome.

Within this framework, inclusion, social support, professional development, financial safety, and autonomy are conceptualized primarily as job resources; work intensity is conceptualized as a job demand; workplace harassment, work-related psychosomatic strain, and health risks are conceptualized as psychosocial or occupational risk-related dimensions; and subjective well-being is conceptualized as the outcome-related psychological dimension.

Some conceptual proximity between dimensions was expected because psychosocial work environment constructs are theoretically interrelated. For example, inclusion and social support both reflect positive social aspects of the work environment, yet they represent distinct constructs: inclusion refers to belonging, participation, and involvement in organizational processes, whereas social support refers to emotional and instrumental support received from colleagues and supervisors. Similarly, work-related psychosomatic strain and health risks are both risk-related dimensions, but the former reflects perceived strain symptoms associated with work, while the latter reflects exposure to physical or occupational health risks. These distinctions informed the theoretical specification of the measurement model and were subsequently evaluated through confirmatory factor analysis and discriminant validity assessment.

### 1.1. Study Aim

The aim of this study was to develop and conduct the initial validation of the Multidimensional Psychosocial Work Environment Scale for Employed Persons (MPWES) and to evaluate its theoretically defined factor structure and psychometric properties in a diverse sample of employed adults in Latvia.

### 1.2. Study Objectives

1. To identify and theoretically define the key dimensions of psychosocial work environment and subjective well-being among employed persons based on the OECD job quality framework, the WHO-5 conceptual approach, and the Job Demands–Resources (JD–R) model.
2. To develop an initial pool of theoretically grounded items reflecting psychosocial work environment factors, psychosocial risks, job resources, and subjective well-being dimensions relevant to occupational settings.
3. To evaluate the content validity of the initial item pool using expert assessment procedures and the Content Validity Index (CVI).
4. To assess the face validity and comprehensibility of the instrument using the Face Validity Index (FVI).
5. To examine the adequacy of the theoretically specified measurement model using confirmatory factor analysis (CFA) as part of the initial validation process.
6. To evaluate discriminant validity of the latent dimensions based on inter-factor correlations.
7. To assess convergent validity and construct reliability using Average Variance Extracted (AVE) and Composite Reliability (CR).
8. To evaluate the internal consistency reliability of the latent dimensions using Cronbach’s alpha and McDonald’s omega.
9. To assess item-level performance using the Response Index (RI) and Discrimination Index (DI).
10. To iteratively refine the instrument by evaluating and revising items and dimensions based on theoretical considerations and empirical findings in preparation for subsequent large-scale validation studies.

## 2. Rationale and Theoretical Framework for the MPWES

### 2.1. Rationale for the Development of the MPWES

In recent decades, psychosocial work environment and employee subjective well-being have become increasingly important topics in occupational health and organizational research. Previous studies have demonstrated that psychosocial working conditions, including job demands, job resources, and workplace psychosocial risks, substantially influence employees’ subjective well-being, mental health, work engagement, and workforce sustainability [1,2,3]. Consequently, the assessment of psychosocial work environment factors has become highly relevant for both occupational health research and organizational practice.

Several validated theoretical frameworks and assessment instruments are currently available for evaluating specific aspects of the psychosocial work environment and employee well-being, including the Job Demands–Resources (JD-R) model [1], the WHO-5 Well-Being Index [7], the PERMA model [5], the OECD job quality framework [8], and the Copenhagen Psychosocial Questionnaire (COPSOQ) [2]. These instruments and frameworks provide valuable insights into psychosocial risks, workplace resources, emotional well-being, and psychological functioning. However, these frameworks and instruments differ in their primary aims, scope, and intended use; some provide broad assessments of the psychosocial work environment, whereas others focus primarily on subjective well-being or on theoretical explanations of work-related processes. This creates a methodological and practical gap, as organizations often need to understand not only whether employees report lower well-being, but also which workplace resources, demands, or psychosocial risks may be associated with this outcome.

For example, WHO-5 primarily assesses emotional subjective well-being [7], whereas COPSOQ focuses on psychosocial working conditions and workplace psychosocial risks [2]. Similarly, the JD-R model conceptualizes job demands and job resources as factors influencing employee health and motivation, but it is not intended to serve as a psychometric instrument for the integrated assessment of the psychosocial work environment [1]. Consequently, organizations and researchers frequently rely on multiple separate instruments to evaluate psychosocial work environment factors and employees’ subjective well-being simultaneously. This may complicate data integration, increase respondent burden, and reduce the practicality of psychosocial risk monitoring and organizational assessment across occupational settings.

The rationale for developing the MPWES was therefore not to duplicate existing instruments, but to provide an integrated, theory-driven, and practically applicable assessment framework in which psychosocial work environment factors and employees’ subjective well-being can be evaluated within a single measurement structure. Administering several established instruments together may provide broad coverage but also increase respondent burden, produce overlapping indicators, complicate score interpretation, and make it difficult to integrate findings into a coherent organizational profile. In contrast, the MPWES was designed to organize key workplace resources, demands, psychosocial risks, and subjective well-being within a single, multidimensional framework, allowing these domains to be interpreted together rather than as separate, disconnected measures.

In addition, several existing instruments are relatively extensive, focused on specific occupational groups, or designed to assess isolated psychosocial risks rather than to support integrated psychosocial work environment screening across different employment sectors [8]. As interest in employee mental health, workplace sustainability, and ESG-related social indicators continues to grow, there is a need for a multidimensional, practically applicable instrument that integrates key psychosocial work environment factors and dimensions associated with employees’ subjective well-being.

Previous studies conducted in Latvia have demonstrated that healthcare professionals’ subjective well-being is substantially influenced by psychosocial work environment factors, including work intensity, emotional strain, autonomy, and organizational ethical climate [10,12]. Furthermore, sectoral differences in psychosocial risks and job resources have been identified across healthcare, pharmaceutical, administrative, and energy sectors [11]. These findings highlight the importance of integrated psychosocial work environment assessment approaches that can identify psychosocial risks, workplace resources, and factors associated with employees’ subjective well-being across occupational contexts.

Therefore, the present study introduces the Multidimensional Psychosocial Work Environment Scale for Employed Persons (MPWES). The instrument was developed based on the Job Demands–Resources (JD-R) model, the OECD job quality framework, and conceptual principles of subjective well-being. MPWES was designed as an integrated psychosocial work environment assessment instrument that incorporates dimensions of job demands, job resources, psychosocial risks, and subjective well-being, while conceptually distinguishing workplace factors from subjective well-being as a psychological outcome. Accordingly, subjective well-being is conceptualized as an outcome-related psychological dimension, whereas the remaining MPWES dimensions represent psychosocial work environment factors that may function as resources, demands, or risks within the occupational context.

Within this conceptual structure, Inclusion, Social Support, Professional Development, Financial Safety, and Autonomy are primarily interpreted as job resources, because they reflect supportive, protective, or enabling aspects of the work environment. Work Intensity is interpreted as a job demand, as it reflects workload, time pressure, and intensive work requirements. Workplace Harassment, Work-related Psychosomatic Strain, and Health Risks are interpreted as psychosocial or occupational risk-related dimensions because they capture exposure to adverse workplace experiences, strain symptoms, or occupational health risks. Subjective Well-Being is therefore not treated as another workplace exposure but as a psychological dimension theoretically associated with these work-environment factors.

Unlike existing instruments, MPWES is not intended solely to assess isolated psychosocial risks or dimensions of emotional well-being. Instead, it was developed as a multidimensional organizational screening instrument to integratively evaluate psychosocial work environment factors and identify potential risks to employees’ subjective well-being across different occupational sectors. Such an approach may provide practical value for occupational health research, psychosocial risk monitoring, workplace prevention strategies, and organizational sustainability initiatives.

The integrated structure of the MPWES is intended to support interpretation of how workplace resources, demands, and risks coexist within the same work environment and how these dimensions are conceptually related to employee subjective well-being.

The theoretical frameworks and instruments informing the development of the MPWES are summarized in Table 1.

Accordingly, the novelty of the MPWES lies not in replacing established instruments such as the WHO-5 or COPSOQ, but in integrating theoretically selected domains from occupational health, job quality, and subjective well-being frameworks into a single preliminary assessment instrument. This integrated structure is intended to support the simultaneous interpretation of psychosocial resources, job demands, risk-related exposures, and employees’ subjective well-being within a single occupational assessment model.

A dimensional comparison between the MPWES and established psychosocial work environment and well-being instruments is presented in Table 2.

As shown in Table 2, the MPWES differs from existing instruments not by introducing entirely new psychosocial domains, but by organizing selected workplace resources, job demands, psychosocial and occupational risk-related dimensions, and subjective well-being within a single theory-driven assessment framework. This structure allows the dimensions to be interpreted together as part of an integrated psychosocial work environment profile. Therefore, the added value of the MPWES lies in its integrative organization and intended simultaneous interpretation rather than in replacing broader established instruments such as COPSOQ-III or QPSNordic.

### 2.2. Conceptual Framework of the MPWES

The MPWES was developed based on three complementary theoretical and conceptual frameworks relevant to psychosocial work environment assessment and employee subjective well-being:

- OECD Job Quality Framework—emphasizes job quality, workplace conditions, organizational environment, and work-related quality of life, supporting multidimensional occupational assessment across employment sectors [8].
- WHO-5 Well-Being Index—focuses on emotional subjective well-being and psychological functioning within health and occupational contexts [7].
- The Job Demands–Resources (JD-R) model conceptualizes how job demands and job resources influence employee strain, motivation, health, and occupational functioning [1].

In addition to these three primary theoretical frameworks, the Copenhagen Psychosocial Questionnaire (COPSOQ) was used as a methodological and comparative reference rather than as a primary theoretical framework. COPSOQ informed the identification of relevant psychosocial work environment domains and supported the conceptualization of item content related to workplace demands, resources, and psychosocial risks. However, the MPWES was not developed as a shortened version or direct adaptation of COPSOQ. Instead, COPSOQ served as one of several established reference instruments to ensure that the MPWES item pool covered relevant psychosocial work-environment content while remaining theoretically organized around the JD-R model, the OECD job quality framework, and principles of subjective well-being.

These frameworks were selected because they:

1. Provide complementary perspectives relevant to psychosocial work environment assessment and subjective well-being in occupational settings.
2. Include dimensions related to job demands, job resources, workplace conditions, psychosocial risks, and emotional well-being.
3. They are widely applied and empirically supported in occupational health and organizational research.
4. Support multidimensional assessment of psychosocial workplace factors across different occupational settings.
5. Facilitate integrated psychosocial risk monitoring and organizational assessment of factors associated with employee subjective well-being.

The conceptual framework of the MPWES is based on the distinction between psychosocial work environment factors and subjective well-being.

It is important to clarify the status of Subjective Well-Being within the MPWES framework. The MPWES is not specified as a causal structural model in which psychosocial work environment factors are tested as predictors of subjective well-being. Rather, it is conceptualized as a mixed, multidimensional screening instrument that includes both psychosocial work-environment dimensions and an outcome-related subjective well-being dimension within the same assessment framework.

In the confirmatory factor analysis, Subjective Well-Being was modeled as a latent measurement dimension to evaluate whether its items formed a coherent factor within the overall MPWES structure. This does not imply that Subjective Well-Being was treated as a causal outcome in the CFA model. Instead, the term “outcome-related dimension” refers to the theoretical interpretation of Subjective Well-Being as a psychological state that may be associated with characteristics of the psychosocial work environment. Therefore, the MPWES should be interpreted as an integrated preliminary assessment framework rather than as a structural model testing causal pathways between predictors and outcomes.

The selection of the ten MPWES dimensions was based on a theory-driven domain-mapping process. First, relevant psychosocial work environment and well-being domains were identified from the JD-R model, the OECD job quality framework, the WHO-5 conceptual approach, COPSOQ, and previous empirical findings from occupational health research. Second, conceptually overlapping or closely related domains were grouped into broader categories representing job resources, job demands, psychosocial or occupational risk-related dimensions, and subjective well-being. Third, domains were retained if they met three criteria: theoretical relevance, relevance to occupational health and employee well-being, and suitability for assessment within a multidimensional workplace screening instrument.

This process resulted in ten preliminary dimensions: Subjective Well-Being, Inclusion, Social Support, Workplace Harassment, Work Intensity, Work-related Psychosomatic Strain, Professional Development, Health Risks, Financial Safety, and Autonomy. The number of dimensions was therefore not determined statistically at this stage but reflected the outcome of the theory-driven domain-mapping and item-development process. We acknowledge that no formal Delphi procedure was conducted to determine the final domain structure. Therefore, the ten-dimensional structure should be regarded as preliminary and requires further evaluation in future studies using formal consensus methods, exploratory factor analysis, exploratory structural equation modeling, and independent validation samples.

Psychosocial work environment factors are understood as workplace-level conditions, exposures, and resources, whereas subjective well-being is understood as an individual psychological state that may be influenced by these workplace factors. This distinction was used to organize the MPWES dimensions into four conceptual groups: job resources, job demands, psychosocial and occupational risk-related dimensions, and outcome-related subjective well-being.

Job resources include Inclusion, Social Support, Professional Development, Financial Safety, and Autonomy. These dimensions reflect supportive and enabling aspects of the work environment that may help employees cope with work demands and maintain well-being. Job demands are represented by Work Intensity, which reflects workload, time pressure, and intensive work requirements. Psychosocial and occupational risk-related dimensions include Workplace Harassment, Work-related Psychosomatic Strain, and Health Risks. These dimensions capture exposure to adverse social experiences, work-related strain symptoms, and occupational health risks. Subjective Well-Being represents the outcome-related psychological dimension and reflects emotional well-being, positive functioning, and perceived psychological balance within the work context.

Some conceptual proximity between MPWES dimensions was expected because psychosocial work environment constructs are naturally interrelated. However, the dimensions were theoretically distinguished before empirical testing. For example, Inclusion and Social Support both reflect positive social aspects of the work environment, but they represent different constructs. Inclusion refers to perceived belonging, participation, and involvement in organizational processes, whereas Social Support refers to emotional and instrumental support from colleagues and supervisors. Similarly, Work-related Psychosomatic Strain and Health Risks are both risk-related dimensions, but the former reflects psychological or physical strain symptoms associated with work, while the latter reflects exposure to physical, ergonomic, or occupational health risks.

This conceptual differentiation informed the theory-driven specification of the MPWES measurement model. The empirical distinctiveness of the dimensions was subsequently evaluated through confirmatory factor analysis and discriminant validity assessment. Therefore, the MPWES should be understood as an integrated but conceptually differentiated framework for assessing psychosocial work environment factors and their relationship to employee subjective well-being.

**Figure 1:**
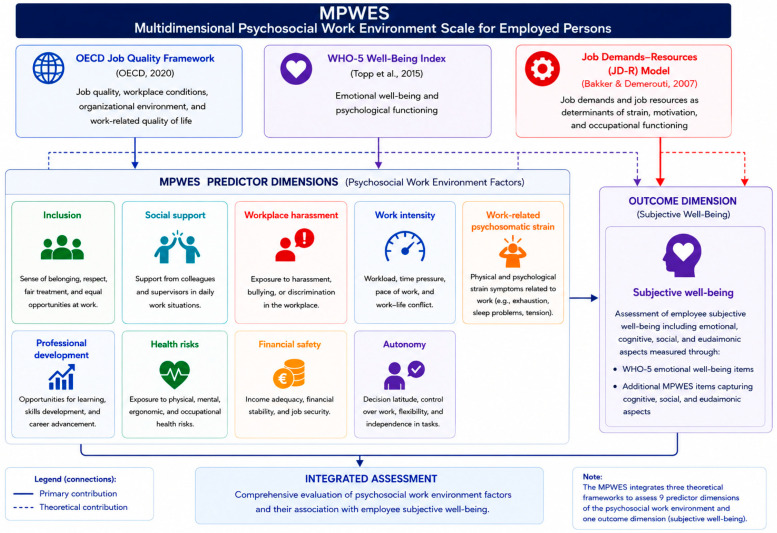
Conceptual structure of the Multidimensional Psychosocial Work Environment Scale for Employed Persons (MPWES), based on the OECD Job Quality Framework, the WHO-5 Well-Being Index, and the Job Demands–Resources model [1,7,8].

The integration of these frameworks and their relationship to the MPWES dimensions are illustrated in Figure 1.

## 3. Development and Initial Validation Process of the MPWES

The development of the Multidimensional Psychosocial Work Environment Scale for Employed Persons (MPWES) followed a systematic, evidence-based, and theory-driven process. The development process integrated established occupational health and psychosocial work-environment frameworks, a targeted literature review, and psychometric validation procedures to ensure conceptual clarity and methodological rigor.

The primary aim of the instrument development process was to create a multidimensional assessment instrument capable of evaluating psychosocial work environment factors, including job demands, job resources, psychosocial risks, workplace conditions, and dimensions associated with employee subjective well-being across occupational settings.

Instrument development was conducted by a multidisciplinary research team comprising occupational health researchers, psychosocial risk assessment specialists, a psychometrician, and a data analyst. All procedures followed internationally recognized recommendations for psychometric instrument development, and ethical principles were observed throughout the study.

**Figure 2:**
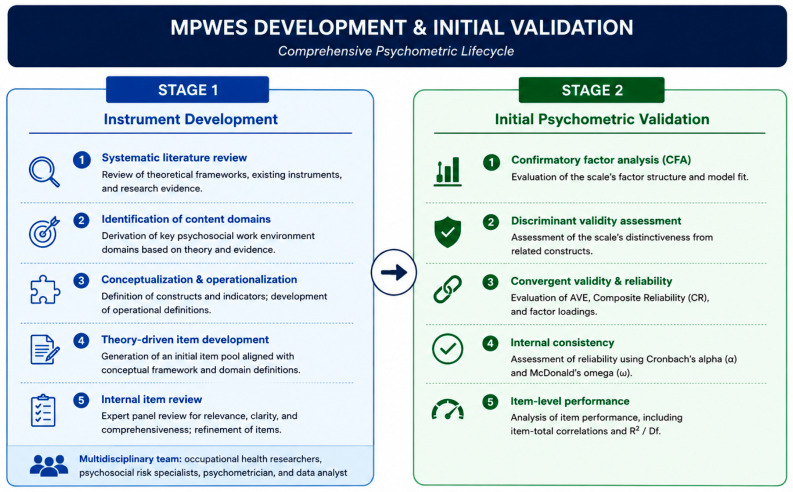
Stages of Development and Initial Validation of the Multidimensional Psychosocial Work Environment Scale for Employed Persons (MPWES).

The development and initial validation process consisted of two major stages, which are summarized in Figure 2.

### 3.1. Stage 1: Scale Development

#### 3.1.1. Targeted Literature Review

A targeted literature review was conducted to identify theoretical frameworks, conceptual models, and existing instruments relevant to psychosocial work environment assessment and employee subjective well-being. The search was conducted in ScienceDirect, Scopus, Web of Science, PubMed, ProQuest, and Wiley Online Library. Search terms included combinations of the following keywords: (“occupational well-being” OR “workplace well-being” OR “subjective well-being”) AND (“psychosocial work environment” OR “job demands” OR “job resources” OR “psychosocial risks”).

The review demonstrated that existing instruments, such as the WHO-5, PERMA, COPSOQ, and JD-R–based assessment approaches, primarily focus on specific dimensions of employee well-being, psychosocial risks, or workplace conditions. However, these instruments and frameworks differ in their primary aims, scope, and intended use. Therefore, the review supported the need to develop a concise, theory-driven instrument that integrates selected psychosocial work environment factors with an outcome-related dimension of subjective well-being within a unified preliminary occupational assessment framework. This gap motivated the development of the Multidimensional Psychosocial Work Environment Scale for Employed Persons (MPWES).

#### 3.1.2. Identification of Psychosocial Well-Being Content Domains

A targeted scoping analysis of the OECD Job Quality Framework [8], the WHO-5 Well-Being Index [7], and the Job Demands–Resources (JD-R) model [1] was conducted to identify theoretically relevant psychosocial work environment dimensions and subjective well-being–related dimensions applicable to occupational settings.

Based on this theoretical synthesis, ten a priori theoretically grounded domains were defined. Nine domains represented psychosocial work environment factors, including job demands, job resources, workplace psychosocial risks, and organizational conditions, whereas one domain represented subjective well-being as an outcome-related psychological dimension [1,8].

These domains formed the conceptual foundation for instrument development and ensured multidimensional coverage of psychosocial work environment factors and dimensions that are theoretically associated with employees’ subjective well-being [1,7,8].

Guided by these theoretically specified domains, an initial pool of 45 items was developed to capture diverse work-related experiences, psychosocial exposures, workplace resources, and perceptions related to subjective well-being. The complete English and Latvian versions of the MPWES questionnaire are presented in the Supplementary Materials (Supplementary Files S1 and S2). Each item was explicitly formulated to reflect a predefined theoretical domain, resulting in a theoretically specified domain–item structure, which was subsequently evaluated using confirmatory factor analysis (CFA) [13,14].

Because the MPWES was developed using an explicitly theory-driven approach, the latent structure was specified a priori. Therefore, the purpose of the initial psychometric analysis was to evaluate the adequacy of this predefined domain–item structure rather than to explore an unknown latent structure. For this reason, confirmatory factor analysis (CFA) was selected as the primary structural validation method in the present study.

#### 3.1.3. Conceptualization and Operationalization

During the conceptualization phase, theoretically relevant psychosocial work environment domains and subjective well-being–related dimensions were identified based on three complementary theoretical and conceptual frameworks: the OECD Job Quality Framework, the WHO-5 Well-Being Index, and the Job Demands–Resources (JD–R) model. These frameworks were applied in a complementary rather than hierarchical manner, with each contributing distinct theoretical perspectives relevant to psychosocial work environment assessment and employee subjective well-being within occupational settings.

The OECD Job Quality Framework contributed dimensions related to workplace conditions, organizational environment, financial security, and work-related quality of life [8]. The WHO-5 conceptual approach informed the subjective well-being dimension by emphasizing emotional well-being and psychological functioning [7]. The JD–R model provided the primary occupational framework for conceptualizing psychosocial work environment factors through the interaction between job demands and job resources [1].

In the operationalization phase, these conceptual definitions were translated into measurable item-level indicators. The research team established explicit coding principles to guide the transformation of theoretical constructs into empirically assessable questionnaire items. For example, subjective well-being was operationalized using indicators of emotional well-being and positive psychological functioning; interpersonal trust, using perceptions of social support and inclusion; and psychosocial risk exposure, using indicators of workplace harassment and work-related psychosomatic strain.

Within this process, theoretically defined workplace factors were systematically categorized according to the JD–R framework. Constructs reflecting supportive working conditions, autonomy, professional development opportunities, inclusion, and social support were conceptualized as job resources, whereas indicators related to workload, time pressure, emotional strain, and intensive work demands were classified as job demands [1]. Workplace harassment, health-related risks, and work-related psychosomatic strain were conceptualized as psychosocial risk–related dimensions associated with occupational stress exposure.

Attention was paid to ensure that all item formulations were conceptually clear, behaviorally interpretable, contextually relevant to occupational settings, and suitable for subsequent psychometric assessment. This structured mapping process ensured conceptual coherence between the theoretical domains and their corresponding item indicators, yielding a theoretically specified domain–item structure that served as the basis for the subsequent confirmatory factor analysis (CFA).

#### 3.1.4. Theory-Driven Item Development

To illustrate the theory-driven domain mapping process, Table 3 presents selected psychosocial work environment domains and subjective well-being–related dimensions together with illustrative item examples. These examples were generated during the initial stage of instrument development to demonstrate how theoretically defined domains were translated into potentially measurable item formulations relevant to occupational settings.

Importantly, the presented examples do not represent the final wording of the instrument items. Rather, they are intended to illustrate the conceptual linkage between predefined theoretical domains and their corresponding item indicators. The resulting domain–item structure was subsequently evaluated empirically using confirmatory factor analysis (CFA).

#### 3.1.5. Internal Initial Item Review

The research team conducted an internal review to ensure conceptual clarity, linguistic precision, contextual relevance, and overall coherence of the preliminary item formulations. Each item was evaluated for readability, avoidance of ambiguous or overly technical language, grammatical consistency, and alignment with the underlying theoretical domains guiding instrument development.

Attention was paid to ensuring that the items were behaviorally interpretable, applicable across occupational settings, and conceptually consistent with the psychosocial work environment framework underlying the MPWES.

Following the internal review process, all items were retained, though several formulations were refined to improve clarity and comprehensibility. As a result, Stage 1 yielded a theoretically grounded, conceptually coherent, and linguistically refined preliminary 45-item version of the Multidimensional Psychosocial Work Environment Scale for Employed Persons (MPWES), which subsequently proceeded to expert content and face validity assessment (CVI and FVI) and further psychometric evaluation in Stage 2.

### 3.2. Stage 2: Initial Psychometric Validation

#### 3.2.1. Expert Content Validity Assessment Using the Content Validity Index (CVI)

##### Expert Panel Selection

Content validity assessment was conducted in accordance with the methodological recommendations of Lynn [15] and Polit and Beck [16], to evaluate the conceptual relevance, clarity, and representativeness of the preliminary 45-item MPWES item pool prior to empirical psychometric testing.

##### Selection Criteria

Six independent experts were recruited based on the following criteria:

- at least five years of professional experience in psychosocial work environment assessment, occupational health, human resource management, organizational processes, or psychometric evaluation;
- no prior involvement in the instrument development process to minimize potential bias;
- professional expertise relevant to the assessment of psychosocial work environment factors and employee subjective well-being in occupational settings.

##### Panel Composition

Six experts participated in the content validity assessment, ensuring both professional and gender balance (three women and three men). This number satisfies established methodological recommendations for CVI assessment and allows reliable estimation of item-level content validity indices [15].

The expert panel consisted of a data analyst, two healthcare professionals, a psychologist, a pharmacist, and an occupational safety specialist. The panel was intentionally multidisciplinary because the MPWES was designed to assess psychosocial work environment factors across different occupational contexts rather than within a single professional group.

The psychologist contributed expertise in subjective well-being, psychological functioning, psychosocial assessment, and the conceptual interpretation of workplace-related psychological dimensions. The healthcare professionals contributed expertise in occupational strain, workplace demands, health-related work conditions, and employee well-being in high-demand service settings. The occupational safety specialist contributed expertise in workplace risk assessment, occupational hazards, and the practical applicability of items related to health risks and safety-sensitive working conditions.

The pharmacist contributed professional expertise regarding regulated healthcare and pharmaceutical work environments, including responsibility, work intensity, client-oriented service demands, professional development, and health-related workplace risks. The data analyst contributed methodological expertise on item clarity, response formats, data structure, variable coding, and the suitability of item formulations for subsequent quantitative analysis.

Although not all panel members were psychometricians, each expert contributed relevant theoretical, methodological, or applied expertise for evaluating the content relevance, clarity, occupational applicability, and practical interpretability of the preliminary MPWES item pool. This multidisciplinary composition supported the assessment of both the conceptual relevance and the practical applicability of the items across different employment settings.

##### Rating Procedure

Content validity assessment followed the methodological procedures proposed by Lynn [15] and Polit, Beck, and Owen [16]. Each expert received a calibration document containing operational definitions of conceptual relevance together with annotated scoring examples to promote consistent interpretation of the evaluation criteria.

Item relevance was assessed using a four-point ordinal rating scale ranging from 1 (“not relevant”) to 4 (“highly relevant”). In accordance with established psychometric recommendations, ratings were dichotomized for CVI analysis, with scores of 3–4 classified as relevant and 1–2 as not relevant.

Content validity indices were calculated at both the item level (I-CVI) and the scale level using the average method (S-CVI/Ave). To account for potential chance agreement among experts, the modified kappa coefficient (κ*) was additionally calculated following the procedure described by Polit, Beck, and Owen [16].

Item retention decisions were guided by established psychometric criteria. Items with I-CVI values ≥ 0.83 were retained; values between 0.70 and 0.79 indicated the need for item revision, and values below 0.70 resulted in item exclusion [15]. These procedures ensured that only conceptually relevant and occupationally interpretable items progressed to subsequent stages of MPWES development and psychometric evaluation.

#### 3.2.2. Face Validity Assessment Using the Face Validity Index (FVI)

##### Participant Selection

Following the expert-based content validity assessment, face validity was evaluated among representatives of the target population to assess the perceived clarity, comprehensibility, interpretability, and occupational relevance of the preliminary MPWES items from the perspective of potential respondents.

##### Selection Criteria

Participants included in the face validity assessment were selected according to the following criteria:

- current employment in either full-time or part-time work;
- employment within one of the occupational sectors included in the main study (healthcare, pharmaceutical, energy, or administrative sectors);
- sufficient proficiency in the Latvian language to ensure accurate understanding and interpretation of the item formulations;
- no prior involvement in the instrument development process or in the expert-based content validity assessment.

These criteria were applied to ensure that the face validity assessment reflected the perspectives of the target population while minimizing potential bias and focusing specifically on item clarity, interpretability, and contextual relevance to occupational settings.

##### Participants

The face validity assessment was conducted with five employed participants purposively selected from the target population to ensure representation across the occupational sectors included in the main study.

The participant group included two respondents from the healthcare sector, one from the pharmaceutical sector, one from the energy sector, and one from the administrative sector.

All participants were employed, either full-time or part-time, and met the inclusion criteria of the main study. None of the respondents had previously participated in the instrument development process or in the expert-based content validity assessment.

##### Rating Procedure for Face Validity (FVI)

Face validity assessment focused on the evaluation of linguistic clarity, comprehensibility, interpretability, and contextual appropriateness of the preliminary item formulations. Participants received standardized instructions together with operational definitions of item clarity and illustrative rating examples to support consistent interpretation of the evaluation criteria.

Item clarity was evaluated using a dichotomous response format (1 = clear; 0 = unclear), which served as the basis for calculating the Face Validity Index (FVI). Face validity indices were calculated at both the item level (I-FVI) and the scale level using the average method (S-FVI/Ave).

In accordance with established methodological recommendations [16], items with I-FVI values ≥ 0.80 were considered sufficiently clear, values between 0.60 and 0.79 indicated the need for linguistic refinement, and values below 0.60 resulted in item exclusion.

These procedures ensured that only linguistically clear, contextually interpretable, and occupationally relevant items progressed to the subsequent stages of MPWES psychometric evaluation.

#### 3.2.3. Psychometric Evaluation of the Theoretically Specified Measurement Model (CFA, Discriminant Validity, Convergent Validity, Internal Consistency Reliability, and Item-Level Performance)

##### Participants

The initial psychometric evaluation of the MPWES was conducted among employed adults aged 18 years and older working full- or part-time across four occupational sectors: administration and maintenance services, healthcare, pharmacy, and energy.

These occupational sectors were intentionally selected to ensure variability in psychosocial work environment characteristics, job demands, job resources, workplace conditions, and occupational risk exposure.

Organizations were selected purposively to ensure variation in psychosocial work environment characteristics, occupational demands, organizational structures, and exposure to work-related risks. Eligible organizations were required to employ adult workers, represent one of the predefined occupational sectors, provide access to employees through an organizational contact person, and agree to anonymous voluntary data collection. Organizations were excluded if anonymous participation could not be ensured, if employees were not directly employed within the organization, or if organizational access could not be granted during the data collection period.

The healthcare sector was included due to its high emotional demands, time pressure, and increased exposure to occupational stress and burnout-related risks. The pharmaceutical sector was selected to represent client-oriented, cognitively demanding work environments characterized by high responsibility and stringent regulatory requirements. The energy sector was included to reflect physically demanding and safety-sensitive occupational settings associated with elevated occupational health risks and work strain. Administration and maintenance services were incorporated to capture occupational contexts characterized by repetitive work tasks, lower decision latitude, and varying levels of work monotony.

The preliminary MPWES instrument was distributed to 300 employees, of whom 213 completed the assessment, resulting in a response rate of 62.3%. All participants demonstrated sufficient proficiency in the Latvian language to ensure accurate comprehension and interpretation of the instrument items. Data from all 213 respondents were included in the psychometric analyses.

Given the study’s initial validation aim and the theory-driven specification of the measurement structure, the sample size was considered sufficient for a preliminary evaluation of the global model fit but not necessarily for stable estimation of all individual CFA parameters. The analysis focused on evaluating the adequacy, interpretability, and psychometric performance of the predefined multidimensional measurement model rather than on exploring alternative latent structures.

The inclusion of respondents from multiple occupational sectors was intended to maximize variability in psychosocial work environment exposures and occupational contexts, thereby supporting the initial cross-sector applicability and psychometric evaluation of the MPWES.

### 3.3. Analytical Strategy and Reporting Standards

All statistical analyses were conducted in accordance with internationally recognized psychometric and reporting standards for instrument development and psychometric evaluation. Descriptive statistics were summarized using means (M), standard deviations (SD), and 95% confidence intervals (CI). When distributional assumptions were not fully met, medians and interquartile ranges (IQR) were additionally reported to provide distribution-robust indicators of central tendency and variability.

Content validity and face validity were evaluated using the Content Validity Index (CVI) and the Face Validity Index (FVI). Item clarity and conceptual relevance were assessed at both the item and scale levels using the Item-level Content Validity Index (I-CVI), the Scale-level Content Validity Index calculated using the average method (S-CVI/Ave), the Item-level Face Validity Index (I-FVI), and the Scale-level Face Validity Index (S-FVI/Ave). To account for potential chance agreement among evaluators, the probability of chance agreement (Pc) and the modified kappa coefficient (κ*) were additionally calculated following the recommendations [16].

To ensure the adequacy of the confirmatory factor analysis (CFA), the sample size (N = 213) was evaluated relative to the model complexity (df = 900). A post hoc power analysis was conducted according to the RMSEA-based framework proposed by MacCallum, Browne, and Sugawara [17]. Statistical power was estimated for testing a close-fit hypothesis (H_0_: RMSEA ≤ 0.05) against a poor-fit alternative (H_1_: RMSEA = 0.08) at α = 0.05. The results indicated adequate statistical power for evaluating the predefined multidimensional measurement model. However, the RMSEA-based post hoc power analysis should be interpreted cautiously. Adequate statistical power for evaluating global model fit does not necessarily mean that the sample size was sufficient to obtain stable estimates of all individual model parameters. Given the complexity of the proposed CFA model, including 45 items and 10 latent factors, the sample size may limit the precision and stability of standardized factor loadings, inter-factor correlations, residual estimates, and reliability-related parameters. An additional methodological limitation is that the present study did not include an exploratory factor analysis prior to confirmatory factor analysis. Although CFA was selected because the MPWES structure was specified a priori based on theoretical frameworks, including the JD-R model, the OECD Job Quality Framework, and the WHO-5 conceptual approach, the absence of an exploratory phase should be acknowledged. In conventional psychometric scale development, EFA is often used to explore the latent structure before confirming it in an independent sample. Therefore, future studies should examine the MPWES using exploratory factor analysis, exploratory structural equation modeling, and confirmatory factor analysis in larger independent samples to further evaluate the stability and replicability of the proposed factor structure. This approach is consistent with confirmatory validation logic, in which item–domain relationships are theoretically specified before empirical testing.

This limitation is particularly relevant for short subscales and dimensions showing estimation instability, such as Financial Safety. Therefore, the CFA findings should be interpreted as preliminary and should be replicated in larger independent samples. Power analyses were performed using the semPower package in R (version 4.5.2).

Confirmatory factor analysis (CFA) was conducted to evaluate the psychometric properties and the hypothesized multidimensional structure of the Multidimensional Psychosocial Work Environment Scale for Employed Persons (MPWES). All analyses were performed using the R statistical environment (version 4.5.2) and the lavaan package. The dataset comprised mixed measurement formats, including six-point Likert-type items and dichotomous indicators. Likert-type items were modeled as reflective indicators of latent psychosocial work environment dimensions and subjective well-being–related dimension, whereas dichotomous indicators represented the presence or absence of psychosocial work environment risks or job-related resources.

Given the categorical, non-normally distributed nature of the data, model estimation was performed using the Weighted Least Squares Mean and Variance adjusted (WLSMV) estimator, which is appropriate for CFA models with ordinal and binary indicators.

Missing data accounted for less than 5% of responses, and no systematic patterns of missingness were identified. Given the low proportion of missing data and the use of the WLSMV estimator for ordinal and binary indicators, missing responses were handled using pairwise deletion within the WLSMV framework. This approach was considered acceptable for the present initial validation study because the amount of missing data was minimal and did not show evidence of systematic missingness. Although multiple imputation and full information maximum likelihood are often recommended in many missing-data contexts, FIML is not directly implemented in the same way for WLSMV estimation with categorical indicators in lavaan. Therefore, pairwise deletion was used as a pragmatic approach compatible with the selected estimator. Nevertheless, this decision represents a methodological limitation, and future validation studies with larger samples should examine the robustness of MPWES findings using alternative missing-data approaches, including multiple imputation where appropriate.

Prior to analysis, data were cleaned and preprocessed, including reverse-coding negatively worded items to ensure directional consistency across latent domains. Model fit was evaluated using multiple robust fit indices, including the Comparative Fit Index (CFI), Tucker–Lewis Index (TLI), Root Mean Square Error of Approximation (RMSEA), and Standardized Root Mean Square Residual (SRMR). Standardized factor loadings ≥ 0.50 were considered acceptable for initial psychometric evaluation.

Discriminant validity was assessed by examining inter-factor correlations. Internal consistency reliability was evaluated using Cronbach’s alpha (α) and McDonald’s omega (ω), together with 95% confidence intervals. For dimensions represented by dichotomous indicators or limited item numbers, reliability coefficients were interpreted cautiously, as these dimensions were conceptualized primarily as occupational exposure–related constructs rather than fully reflective latent variables.

Convergent validity and construct reliability were additionally evaluated using Average Variance Extracted (AVE) and Composite Reliability (CR) based on standardized CFA loadings. Item-level performance was assessed using the Response Index (RI) and the Discrimination Index (DI) to evaluate response distributions and item discrimination performance.

All inferential statistical tests were two-tailed, with statistical significance set at *p* < 0.05.

#### Use of AI-Assisted Tools

ChatGPT Plus (OpenAI, GPT-4) and Grammarly Premium were used solely for language refinement, grammar checking, and stylistic editing of the manuscript. These tools were not used for study design, data collection, data analysis, interpretation of results, or generation of scientific conclusions. All authors reviewed and approved the final text and take full responsibility for the content, accuracy, and scientific integrity of the work.

### 3.4. Ethics Approval and Consent to Participate

The study was conducted in full accordance with the ethical principles of the Declaration of Helsinki. Prior to participation, all respondents were provided with an electronic informed consent form outlining the purpose of the study, the voluntary nature of participation, and the right to withdraw at any time without any consequences. Information consent was obtained from all participants before data collection commenced.

Complete anonymity was ensured, and no personally identifiable information was collected or processed. The study received ethical approval from the Research Ethics Committee of Riga Stradiņš University (Decision No. 2-PEK-4/495/2024).

## 4. MPWES Response Formats and Preliminary Interpretation Framework

The Multidimensional Psychosocial Work Environment Scale for Employed Persons (MPWES) is a multidimensional assessment instrument designed to evaluate psychosocial work environment factors and dimensions associated with employee subjective well-being. Within the MPWES framework, subjective well-being represents an outcome-related psychological dimension, whereas the remaining dimensions assess psychosocial work environment characteristics, including job demands, job resources, psychosocial risks, and organizational conditions that are theoretically associated with employee well-being in occupational settings.

### Conceptual Basis for Response Format Selection

The selection of response formats within the MPWES was guided by theoretical, methodological, and psychometric considerations regarding the most appropriate measurement approach for each domain. Different response formats were intentionally used to reflect the conceptual characteristics of the measured constructs.

Likert-type and temporal frequency scales were used for dimensions conceptualized as continuous psychosocial experiences, perceptions, or workplace conditions. These response formats allow assessment of variability in perceived psychosocial work environment exposures and subjective well-being–related experiences.

Given the absence of universally established normative cut-off values for psychosocial work environment assessment instruments, the MPWES applies comparatively lower, moderate, and higher levels (comparatively lower, moderate, and higher levels of the measured construct) exclusively for descriptive and interpretive purposes during the initial validation stage.

By contrast, dichotomous response formats were used for dimensions representing the presence or absence of specific psychosocial exposures, workplace risks, or access to job resources. These indicators were not conceptualized as continuous latent constructs and were therefore interpreted using binary classifications.

The application of mixed response formats was intended to preserve conceptual consistency across dimensions while supporting practical interpretability in occupational and psychosocial work-environment assessment contexts. The response formats and preliminary interpretation categories for each MPWES dimension are presented in Table 4.

## 5. Results

This section summarizes the results of the three main stages of the initial validation of the Multidimensional Psychosocial Work Environment Scale for Employed Persons (MPWES):

1. Expert content validity assessment using the Content Validity Index (CVI);
2. Face validity assessment using the Face Validity Index (FVI);
3. Psychometric evaluation of the theoretically specified multidimensional measurement model, including:
  3.1 Examination of model fit using confirmatory factor analysis (CFA);
  3.2 Assessment of discriminant validity based on inter-factor correlations;
  3.3 Assessment of convergent validity using Average Variance Extracted (AVE) and Composite Reliability (CR);
  3.4 Evaluation of internal consistency reliability using Cronbach’s alpha and McDonald’s omega;
  3.5 item-level performance analysis using the Response Index (RI) and Discrimination Index (DI).

The initial validation process incorporated three distinct samples of respondents and experts, thereby providing a multidimensional basis for evaluating the conceptual adequacy, psychometric performance, and preliminary applicability of the MPWES across occupational settings. Detailed findings for each validation stage are presented in the corresponding subsections below.

### 5.1. Expert Content Validity (CVI) Assessment

#### 5.1.1. Expert Characteristics

The content validity assessment was conducted by a panel of six independent experts. The panel was gender-balanced and included professionals from multiple disciplines relevant to psychosocial work environment assessment, occupational health, organizational processes, and employee well-being research.

The panel consisted of three women (50%) and three men (50%), with a mean age of 38 years (range: 37–49 years). The experts demonstrated substantial professional experience, with a mean of 10.2 years (SD = 3.0), and all fulfilled the predefined inclusion criterion of at least five years of professional experience in psychosocial work environment assessment, occupational health, human resource management, healthcare, or related fields.

Regarding educational attainment, four experts held master’s degrees (66.7%), whereas two held doctoral degrees (33.3%), indicating a high level of theoretical competence and familiarity with psychometric evaluation and instrument development principles.

The panel represented several professional domains relevant to psychosocial work environment assessment and occupational research, including healthcare (*n* = 2), occupational safety (*n* = 1), data analytics and public health (*n* = 1), psychology (*n* = 1), and pharmacy (*n* = 1).

The multidisciplinary composition of the expert panel was considered appropriate because the MPWES was designed to assess psychosocial work-environment factors across diverse occupational contexts rather than within a single professional group. The psychology expert contributed expertise in subjective well-being, psychological functioning, psychosocial assessment, and the conceptual interpretation of workplace-related psychological dimensions. The healthcare experts contributed professional knowledge regarding occupational strain, workplace demands, health-related work conditions, and employee well-being in high-demand service settings. The occupational safety expert contributed expertise in workplace risk assessment, occupational hazards, safety-sensitive work conditions, and the practical relevance of health-risk-related items.

The pharmacy expert contributed applied professional knowledge regarding regulated healthcare and pharmaceutical work environments, including responsibility, work intensity, client-oriented service demands, professional development, and health-related workplace risks. Experts in data analytics and public health contributed methodological expertise related to item clarity, response structure, variable coding, data quality, and the suitability of item formulations for subsequent quantitative analysis. Although not all panel members were psychometricians, each expert contributed relevant theoretical, methodological, or applied expertise for evaluating item relevance, clarity, occupational applicability, and practical interpretability.

This multidisciplinary composition enabled evaluation of conceptual relevance, occupational applicability, methodological clarity, and practical interpretability from multiple professional perspectives, thereby strengthening the overall content validity assessment of the MPWES.

#### 5.1.2. Initial Content Validity Assessment

The first round of the content validity assessment indicated that the preliminary 45-item version of the Multidimensional Psychosocial Work Environment Scale for Employed Persons (MPWES) demonstrated strong conceptual relevance and satisfactory content adequacy at the item level. Content validity was evaluated using a structured expert-review procedure based on the Content Validity Index (CVI). Six independent experts evaluated each item using a four-point relevance scale. Ratings of 1 (“not relevant”) and 2 (“somewhat relevant”) were classified as not relevant, whereas ratings of 3 (“quite relevant”) and 4 (“highly relevant”) were classified as relevant to the theoretically defined domain (see Supplementary Material, Table S1).

For CVI calculation, the four-point relevance scale was dichotomized by collapsing ratings of 3 and 4 into the relevant category and ratings of 1 and 2 into the not relevant category (see Supplementary Material, Table S2). This procedure is consistent with established methodological recommendations for content validity assessment and enables standardized aggregation and interpretation of expert evaluations [15,16].

Based on this classification approach, the Item-level Content Validity Index (I-CVI) was calculated for each item, and the resulting item- and scale-level CVI indices are summarized in Table 5.

In addition to the I-CVI, the probability of chance agreement (Pc) and the modified kappa coefficient (κ*) were calculated for each item to determine whether the observed level of expert agreement exceeded that expected by chance alone. Incorporating Pc and κ* provided a more conservative and methodologically rigorous interpretation of item-level content validity and reduced the risk of overestimating expert agreement (see Table 5).

#### 5.1.3. Item-Level Results

Item-level content validity was evaluated using the Item-level Content Validity Index (I-CVI), complemented by the probability of chance agreement (Pc) and the modified kappa coefficient (κ*) to assess expert agreement beyond chance expectations. As shown in Table 5, I-CVI values from the first round of expert evaluation ranged from 0.67 to 1.00, indicating generally high levels of expert consensus on the conceptual relevance and representativeness of the preliminary MPWES items.

Most items achieved an I-CVI of 1.00, indicating unanimous agreement among all six experts on item relevance. Several items demonstrated an I-CVI of 0.83, indicating good content validity and agreement among five experts. These included Item 22 (Subjective well-being), Items 16 and 18 (Inclusion), Items 8 and 12 (Social support), Items 36 and 38 (Workplace harassment), Items 26 and 29 (Work intensity), and Item 27 (Autonomy).

A smaller subset of items yielded an I-CVI value of 0.67, indicating agreement among four experts and comparatively lower perceived content relevance. These included Item 6 (Subjective well-being), Item 35 (Workplace harassment), Items 39 and 40 (Work-related psychosomatic strain), Item 24 (Health risks), and Items 10 and 11 (Financial safety). These items were subsequently identified as priorities for conceptual clarification and linguistic refinement prior to further psychometric evaluation.

The probability of chance agreement (Pc) varied according to the number of experts endorsing item relevance. Lower Pc values were observed for items demonstrating unanimous agreement (Pc = 0.016), whereas higher Pc values were identified for items endorsed by five experts (Pc = 0.094) and four experts (Pc = 0.234). Accordingly, modified kappa coefficients (κ*) ranged from 0.57 to 1.00. Excellent agreement was observed for items with unanimous expert endorsement (κ* = 1.00), whereas lower, though still acceptable, agreement levels were observed for items endorsed by four experts.

Collectively, the I-CVI, Pc, and κ* results provide consistent evidence of strong preliminary item-level content validity while simultaneously identifying several items requiring refinement before subsequent stages of psychometric evaluation.

#### 5.1.4. Domain-Level Content Validity Findings

Scale-level content validity was evaluated using the Scale-level Content Validity Index (S-CVI/Ave) calculated by the average method. As shown in Table 5, S-CVI/Ave values obtained during the first round of expert evaluation varied across the theoretical domains, reflecting differing levels of expert consensus within each domain.

Several domains demonstrated excellent scale-level content validity, including Subjective well-being (S-CVI/Ave = 0.95), Inclusion (0.96), Social support (0.96), Professional development (1.00), Work intensity (0.92), and Autonomy (0.92). These findings indicate strong expert agreement on the relevance and conceptual representativeness of the items in these domains.

Moderate to good scale-level content validity was observed for Health risks (S-CVI/Ave = 0.84) and Workplace harassment (S-CVI/Ave = 0.79), suggesting acceptable overall domain representation, with several items identified for further refinement. Lower scale-level content validity was observed for Work-related psychosomatic strain (S-CVI/Ave = 0.67) and Financial safety (S-CVI/Ave = 0.67), identifying these domains as priorities for targeted conceptual clarification and linguistic refinement subsequent psychometric evaluation.

#### 5.1.5. Overall Instrument-Level Content Validity

In addition to item- and domain-level analyses, an overall instrument-level Content Validity Index was calculated using the scale-level average method (S-CVI/Ave). The overall instrument-level CVI reached 0.90 (see Supplementary Material Table S2), indicating strong preliminary content validity of the Multidimensional Psychosocial Work Environment Scale for Employed Persons (MPWES) and exceeding commonly recommended thresholds for studies conducted with six experts [15,16].

This finding reflects a high degree of expert agreement regarding item relevance across the instrument while accommodating moderate variability across several theoretical domains. Collectively, these results provide a robust methodological foundation for subsequent stages of psychometric evaluation, including construct validity assessment and structural model evaluation.

#### 5.1.6. Item Revision and Second-Round Assessment

The item revision process was conducted collaboratively by the research team and was not based on a unilateral decision by a single author. First, the research team reviewed the quantitative I-CVI results together with the experts’ written comments. Second, items requiring revision were discussed in relation to their predefined theoretical domain, conceptual clarity, linguistic precision, and occupational applicability. Third, revised wording was agreed upon by consensus among the authors before the items were resubmitted to the expert panel for Round 2 evaluation. No changes were made to the underlying theoretical domains during this process; revisions were limited to improving item clarity, conceptual alignment, and interpretability.

Based on the expert panel’s qualitative feedback, all items that showed low or non-unanimous expert agreement in Round 1 were conceptually and linguistically refined to improve clarity, precision, and interpretability. The revised items were subsequently resubmitted for reassessment in Round 2, including Items 6 and 22 (Subjective well-being), Items 16 and 18 (Inclusion), Items 8 and 12 (Social support), Items 35, 36, and 38 (Workplace harassment), Items 26 and 29 (Work intensity), Items 39 and 40 (Work-related psychosomatic strain), Item 24 (Health risks), Items 10 and 11 (Financial safety), and Item 27 (Autonomy).

The second-round assessment was conducted with the same panel of six experts, ensuring methodological continuity and enabling direct evaluation of whether the revisions adequately addressed the issues identified in the first-round expert review. Following revision, all reassessed items achieved unanimous expert agreement regarding relevance (I-CVI = 1.00). Details of the item revisions made after Round 1 are presented in Supplementary Material Table S3.

The increase in I-CVI values in Round 2 should therefore be interpreted as reflecting improved item clarity and conceptual alignment after expert-informed revision rather than as evidence that the evaluation criteria were relaxed. Experts were instructed to re-rate the revised items independently using the same relevance criteria and the same four-point rating scale as in Round 1. Consequently, the revised domains demonstrated excellent scale-level content validity in Round 2 (S-CVI/Ave = 1.00).

Overall, these findings indicate that the preliminary version of the Multidimensional Psychosocial Work Environment Scale for Employed Persons (MPWES) demonstrated satisfactory preliminary content validity, with the identified limitations primarily related to item wording and interpretability rather than conceptual inconsistency. The complete expert agreement achieved following revision should be interpreted as evidence of improved item clarity and content relevance after expert-informed refinement. However, because content validity reflects expert judgment rather than empirical construct validation, these findings were treated as a preliminary basis for subsequent psychometric evaluation rather than as definitive evidence of instrument validity.

### 5.2. Small-Group Respondent Face Validity Assessment

#### 5.2.1. Participant Characteristics

Five employed respondents from the target population participated in the Face Validity Index (FVI) assessment. Participants were selected using purposive sampling to ensure relevance to the instrument’s intended application and conceptual content.

The respondent group represented multiple employment sectors, including healthcare (*n* = 2), pharmaceuticals (*n* = 1), energy (*n* = 1), and administrative services (*n* = 1). This sectoral diversity supported the evaluation of item clarity, comprehensibility, and contextual suitability across different occupational settings.

Participants ranged in age from 29 to 47 years, with a mean age of 38.4 years. Professional work experience ranged from 6 to 22 years, with a mean work experience of 8.2 years, indicating sufficient occupational experience to evaluate the practical relevance and interpretability of the instrument items.

Regarding educational attainment, three respondents had tertiary education (bachelor’s or master’s degree in social sciences, health sciences, or management-related fields), whereas two respondents had secondary vocational education. This educational variability supported assessment of item clarity and comprehensibility across different educational levels within the target population.

All respondents were fluent in Latvian, ensuring adequate understanding of the questionnaire items and enabling accurate evaluation of item clarity, comprehensibility, and semantic interpretability.

#### 5.2.2. Face Validity Assessment

As presented in Table 6, the majority of MPWES items demonstrated strong face validity. Most items achieved an Item-level Face Validity Index (I-FVI) of 1.00, indicating unanimous agreement among all five respondents regarding item clarity and comprehensibility. These findings suggest that the wording of the items was generally clear, understandable, and appropriate for the target population.

A smaller subset of items yielded an I-FVI of 0.80, indicating agreement among four of the five respondents regarding item clarity. These items included Items 1, 2, 4, 6, 10, 14, 21, 22, 27, 28, 33, and 40. Although these values meet commonly accepted thresholds for acceptable face validity, they indicate minor variability in item interpretation and suggest the need for limited linguistic refinement.

Respondent-level face validity indices ranged from 0.91 to 0.98, indicating high and consistent agreement across respondents. At the scale level, the overall Face Validity Index (FVI) reached 0.95, demonstrating strong overall face validity of the instrument.

Collectively, these findings indicate that the MPWES items were clear, comprehensible, and appropriate for the intended target population, thereby providing an appropriate methodological basis for subsequent psychometric evaluation.

Items with an I-FVI value of 0.80 underwent minor linguistic refinement aimed at improving clarity and comprehensibility while preserving the original theoretical meaning of the items. The revised formulations were subsequently reviewed by an additional small group of respondents from the target population, all of whom confirmed the clarity and appropriateness of the revised wording. No substantial concerns regarding item comprehensibility were identified following revision.

### 5.3. Pilot Study: Psychometric Assessment

#### 5.3.1. Participant Characteristics

The study sample comprised 213 employed respondents recruited from four organizations representing different economic sectors: administrative and support services, healthcare, pharmaceutical manufacturing, and energy distribution. Participants were recruited from organizations operating in energy distribution in Latvia (*n* = 69; 32.4%), a pharmaceutical manufacturing company (*n* = 51; 23.9%), a regional healthcare institution (*n* = 49; 23.0%), and administrative and support services (*n* = 44; 20.7%). No incomplete questionnaires were identified, and all responses were retained for analysis.

The inclusion of organizations from multiple sectors was intentional to ensure heterogeneity of work environments and psychosocial exposures, thereby strengthening the methodological robustness of the psychometric evaluation and supporting the preliminary cross-sector applicability of the MPWES. The sectors varied considerably in job demands, organizational structures, occupational risks, and regulatory environments.

All participants were actively employed at the time of data collection, and participation was voluntary. Prior to participation, respondents were informed about the study’s aims, the anonymous and confidential handling of data, and their right to withdraw at any time without adverse consequences. Only respondents who provided informed consent were included in the study.

#### 5.3.2. Model Power and Sample Size Adequacy

The adequacy of the sample size (*N* = 213) for evaluating the hypothesized measurement model was examined using a post hoc power analysis. The analysis indicated high statistical power for detecting potential model misspecifications within the confirmatory factor analysis framework.

Despite the multidimensional structure of the MPWES and the relatively small sample size, the substantial number of model degrees of freedom provided adequate statistical power to detect deviations from close model fit. This structural characteristic supported the estimation and evaluation of the hypothesized ten-factor measurement model.

Overall, these findings suggest that the available sample size was adequate for the initial psychometric evaluation of the proposed multidimensional structure of the MPWES. Consequently, the subsequent confirmatory factor analysis results may be interpreted with an appropriate degree of statistical confidence, while acknowledging the validation process’s preliminary nature.

#### 5.3.3. Confirmatory Factor Analysis Results

Confirmatory factor analysis provided partial support for the hypothesized multidimensional measurement structure of the MPWES. The robust chi-square test was statistically significant (χ^2^(900) = 10,572.10, *p* < 0.001), which is not uncommon in complex multidimensional models, given that the chi-square statistic is sensitive to sample size, model complexity, and the number of estimated parameters [18,19].

The incremental fit indices (CFI = 0.85, TLI = 0.84) indicated a limited-to-moderate model fit and were below the commonly recommended thresholds. Therefore, the CFA results should not be interpreted as evidence of optimal model fit. Rather, they suggest that the proposed ten-factor structure provides a theoretically interpretable but psychometrically preliminary representation of the MPWES measurement model.

The approximate and absolute fit indices (RMSEA = 0.081, SRMR = 0.078) approached commonly accepted thresholds and indicated borderline-to-acceptable fit [20]. Taken together, the fit indices suggest that the hypothesized model is not fully optimized but may be considered acceptable for an initial theory-driven validation study of a complex multidimensional occupational assessment instrument.

Several factors may explain the suboptimal incremental fit indices. First, the MPWES includes ten theoretically distinct but conceptually related dimensions, which increases model complexity. Second, the model includes heterogeneous indicator types, including Likert-type and dichotomous items. Third, several domains are represented by only two or a small number of items and were conceptualized as exposure-oriented or content-driven domains rather than fully reflective latent constructs. Fourth, localized psychometric issues were identified, particularly the weak performance of the Financial Safety dimension and the high inter-factor correlation between Inclusion and Social Support.

Therefore, the current CFA findings should be interpreted as preliminary evidence supporting the theoretical plausibility of the MPWES structure rather than as definitive confirmation of a fully established measurement model. Reliability and convergent validity indices (Cronbach’s α, McDonald’s ω, CR, and AVE) are reported for completeness; however, dimensions represented by two-item or dichotomous indicators should be interpreted cautiously. Further validation in larger and independent samples is required before the ten-factor structure can be considered fully established.

#### 5.3.4. Evaluation of Factor Loadings (CFA)

As summarized in Table 7, most items demonstrated acceptable to strong standardized factor loadings, with most values exceeding the commonly recommended threshold of β ≥ 0.50, indicating adequate item–factor relationships and supporting the proposed multidimensional measurement structure [18,19].

Items with loadings below this threshold were limited in number and construct specific. Specifically, Item 5 within the Social Support factor demonstrated a standardized loading of β = 0.48, whereas Item 11 within the Financial Safety factor showed a loading of β = 0.45. These findings indicate comparatively weaker associations with their respective latent constructs and identify these items as candidates for future refinement and re-evaluation.

In addition, the Financial Safety factor exhibited potential instability in estimation, as Item 10 yielded an excessively high standardized loading (β > 1.00), suggesting possible specification or estimation issues within this domain.

Given this finding, the Financial Safety dimension was not interpreted as a fully established reflective latent construct in the present initial validation study. Instead, it was treated as a preliminary, context-sensitive domain that remains theoretically relevant within the broader psychosocial work environment and job quality framework but requires substantial item revision and further empirical validation.

Overall, the results presented in Table 7 indicate generally satisfactory factor loadings across most MPWES dimensions, while identifying a limited number of items that require targeted psychometric refinement in future validation studies.

#### 5.3.5. Discriminant Validity

Inter-factor correlation analysis indicated that most correlations among the latent factors were weak to moderate, providing overall support for construct distinctiveness while preserving theoretically meaningful associations between related dimensions.

The strongest positive correlations were observed between Inclusion and Social Support (r = 0.88), and between Subjective Well-Being and Social Support (r = 0.69) and Inclusion (r = 0.67). These associations are theoretically plausible and reflect a shared social resource dimension within the occupational context. However, the correlation between Inclusion and Social Support exceeded the commonly cited threshold of 0.85, suggesting potential conceptual overlap and the need for further assessment of discriminant validity between these constructs in subsequent validation stages.

This finding indicates that discriminant validity between Inclusion and Social Support is only partially supported in the present initial validation study. Therefore, these dimensions should be interpreted as theoretically distinct but empirically closely related social resource constructs, and their separation requires further evaluation in future validation studies using additional approaches such as HTMT analysis, exploratory structural equation modeling, and cross-validation in independent samples.

At this stage, the observed overlap was interpreted as theoretically plausible, as both constructs reflect closely related social resource processes within the Job Demands–Resources (JD–R) framework. Inclusion captures perceived belonging and organizational integration, whereas Social Support reflects the availability of emotional and instrumental workplace assistance. Formal discriminant validity assessment using alternative approaches (e.g., HTMT) and cross-validation in independent samples is therefore planned for future validation phases.

Job demand- and risk-related factors (Work Intensity, Workplace Harassment, and Work-related Psychosomatic Strain) demonstrated moderate positive intercorrelations (e.g., Workplace Harassment–Work-related Psychosomatic Strain, r = 0.51), together with negative associations with resource-related constructs, such as Work Intensity with Subjective Well-Being (r = −0.46) and Autonomy (r = −0.18). This pattern of correlations is generally consistent with theoretical expectations derived from the Job Demands–Resources (JD–R) model.

In contrast, Professional Development, Health Risks, and Financial Safety demonstrated weak correlations with other factors (|r| < 0.30), indicating relative construct distinctiveness and acceptable discriminant separation.

Overall, the observed pattern of inter-factor correlations was theoretically coherent and empirically interpretable, supporting the instrument’s proposed multidimensional structure. At the same time, the elevated correlation between Inclusion and Social Support indicates this factor pair as a priority for further evaluation of discriminant validity in future validation studies.

#### 5.3.6. Convergent Validity and Construct Reliability (CR and AVE)

Construct reliability was evaluated using Composite Reliability (CR), whereas convergent validity was assessed using the Average Variance Extracted (AVE). For most factors, CR values exceeded the commonly recommended threshold (CR > 0.70), indicating satisfactory construct reliability.

Overall, convergent validity was acceptable for most constructs, as AVEs were above 0.50, indicating that the latent variables explained more than half of the variance in their indicators. Particularly high AVE values were observed for Workplace Harassment, Work-related Psychosomatic Strain, Professional Development, Health Risks, and Autonomy.

However, the Inclusion (AVE = 0.45) and Social Support (AVE = 0.42) constructs did not reach the recommended AVE threshold, indicating comparatively limited convergent validity. This finding is consistent with previously observed high inter-factor correlations among these constructs and may reflect partial conceptual overlap among related social resource dimensions.

The Financial Safety dimension demonstrated the weakest psychometric performance among the MPWES dimensions, with a very low composite reliability (CR = 0.13) and an inadmissible standardized loading on one item. These findings indicate that Financial Safety should not be interpreted as a stable reflective latent construct in the current version of the MPWES.

Given its theoretical relevance to job quality and perceived economic security, Financial Safety was retained only as a preliminary, index-like, context-sensitive domain at this stage of instrument development. Therefore, reliability and convergent validity indices for this dimension should be interpreted with caution and should not be considered evidence of adequate reflective measurement. Future studies should substantially revise and expand the Financial Safety item pool, evaluate whether this domain is better represented as a formative or index-based component, and determine whether it should be retained or removed from subsequent versions of the MPWES.

Accordingly, these findings should be interpreted in the context of the preliminary, exploratory nature of the current validation stage. Overall, the CR and AVE results support acceptable construct reliability and convergent validity for most MPWES dimensions, while identifying several constructs that require targeted psychometric refinement in future validation studies.

#### 5.3.7. Model Refinement and Sequential CFA Evaluation

Based on the results of the initial confirmatory factor analysis (CFA), the measurement model was refined using a stepwise, sequential procedure to assess the impact of identified psychometric limitations on model fit and structural stability. Following each modification, the model was re-estimated and compared with the original specification using consistent fit evaluation criteria.

The refinement process initially focused on the Financial Safety factor. First, one item was removed, and the model was re-estimated; however, this modification did not result in substantial improvements in global fit indices. Subsequently, both Financial Safety items were removed, and the CFA was rerun; however, this alternative specification also failed to demonstrate meaningful empirical improvement. Considering these findings and the theoretical relevance of financial security within the broader psychosocial work environment framework, Financial Safety was retained only as a preliminary index-like domain in the current version of the MPWES, rather than as a fully established reflective latent factor.

However, retention of the Financial Safety domain should not be interpreted as evidence that this dimension is psychometrically finalized. Rather, the decision reflects the developmental nature of the present study and the theoretical relevance of financial security for psychosocial work environment assessment. The findings indicate that this domain requires substantial conceptual and psychometric refinement before it can be considered a stable reflective component of the MPWES structure.

In the subsequent step, the Social Support factor was examined. Given that Item 5 consistently had standardized factor loadings below the recommended threshold, it was removed, and the model was re-estimated. However, this modification did not substantially improve model fit or meaningfully influence the stability of the Financial Safety factor.

Given the elevated inter-factor correlation between Inclusion and Social Support, these constructs were subsequently combined into a single Social Resources factor, and the model was re-estimated. Nevertheless, this alternative specification did not yield substantial improvements over the original CFA model, either in global fit indices or in construct differentiation.

This finding suggests that although Inclusion and Social Support are empirically highly related, combining them into a single factor did not provide a more convincing or psychometrically superior solution at this stage. Therefore, the two dimensions were retained as separate but closely related social resource constructs in the preliminary MPWES structure. However, the elevated correlation between these factors indicates that their discriminant validity is only partially supported in the present study. Further validation using HTMT analysis, exploratory structural equation modeling, and cross-validation in independent samples is needed to determine whether these dimensions should remain separate or be represented as a broader Social Resources factor in future versions of the instrument.

Overall, the sequential evaluation of alternative model specifications demonstrated that item removal, construct reduction, or factor merging did not provide substantial empirical advantages and, in some cases, reduced theoretical clarity and conceptual coverage. In contrast, the original CFA model demonstrated the most theoretically and empirically interpretable specification, representing an important consideration at the initial stage of scale validation.

Accordingly, the identified limitations may be interpreted as localized areas requiring future refinement rather than indicators of fundamental structural misspecification. These findings support retaining the original measurement model, with further iterative refinement to be addressed in subsequent validation phases.

#### 5.3.8. Reliability and Item Diagnostics

The psychometric quality of the MPWES was evaluated using four complementary indicators. Internal consistency was assessed using Cronbach’s alpha (α) and McDonald’s omega (ω), whereas item functioning was examined using the Response Index (RI) and the Discrimination Index (DI) to evaluate each item’s ability to differentiate between low- and high-scoring respondents [21].

#### 5.3.9. Internal Consistency: Cronbach’s α, α if Item Deleted, and McDonald’s ω

Internal consistency of the MPWES scales was evaluated using Cronbach’s alpha (α) at the factor level, Cronbach’s α if an item was deleted at the item level, and McDonald’s omega (ω) as a complementary reliability estimate.

At the factor level, satisfactory to strong internal consistency was observed for Subjective Well-Being (α = 0.91; ω = 0.91), Inclusion (α = 0.85; ω = 0.85), Social Support (α = 0.81; ω = 0.81), and Work Intensity (α = 0.80; ω = 0.77), indicating strong internal coherence of these scales. Professional Development also demonstrated acceptable reliability (α = 0.78; ω = 0.79).

Lower reliability coefficients were observed for Workplace Harassment (α = 0.67; ω = 0.66) and Financial Safety (α = 0.49). These findings indicate that short subscales and exposure-oriented domains should be interpreted with caution and may require item expansion or reconsideration of their status as reflective constructs in future validation studies. These comparatively lower values are likely attributable to the limited number of items within these factors and, for Financial Safety, to previously identified item-level instability.

Analysis of Cronbach’s α after deleting items demonstrated that removing individual items did not yield meaningful increases in factor-level α for the better-performing scales, supporting item retention within Subjective Well-Being, Inclusion, Social Support, and Work Intensity. In contrast, for factors with lower α coefficients, item-deletion effects were minimal, indicating that reduced reliability was more likely attributable to scale-length constraints than to isolated item-level problems.

For factors consisting of fewer than three items (Work-related Psychosomatic Strain, Health Risks, Financial Safety, and Autonomy), McDonald’s ω and α if an item were deleted were interpreted cautiously, as reliability estimates may become unstable under such conditions (see Table 8).

Overall, the combined α, α if item deleted, and ω findings indicate that the majority of MPWES scales demonstrated satisfactory internal consistency, whereas lower coefficients were primarily attributable to the structural characteristics of short scales rather than to major psychometric limitations.

#### 5.3.10. Response Index (RI)

The Response Index (RI) reflects the average level of item endorsement and provides information regarding response tendencies across individual items. RI values were used to identify potential floor or ceiling effects and to evaluate whether items adequately differentiated between respondents with varying levels of the underlying construct.

For items measured using 6-point Likert-type scales, acceptable RI values ranged from 2 to 5, indicating balanced endorsement levels and appropriate utilization of the response scale. For dichotomous items scored from 1 to 2, the recommended RI range was 1.20–1.80. Lower RI values indicate items that are less frequently endorsed, whereas values approaching or exceeding the upper threshold may reflect highly endorsed items potentially susceptible to ceiling effects [18].

Across the Likert-scaled dimensions Subjective Well-Being, Inclusion, Social Support, Work Intensity, and Autonomy, most RI values fell within the recommended range, indicating adequate endorsement distribution and sufficient response variability. However, within the Social Support factor, several items exceeded the upper RI threshold: Item 3 (RI = 5.10), Item 5 (RI = 5.20), and Item 8 (RI = 5.04). These elevated values suggest potential ceiling effects, indicating that these items were highly endorsed and may therefore demonstrate reduced discriminative variability.

Distinct RI patterns were observed for dichotomous indicators. Within the Professional Development factor (Items 32–34), RI values ranged from 1.29 to 1.38, remaining fully within the recommended interval and indicating balanced endorsement levels with satisfactory differentiation across respondents. Similarly, the Work-related Psychosomatic Strain factor (Items 39–40) demonstrated RI values of 1.57 and 1.75, both within the acceptable range, suggesting balanced endorsement without pronounced floor or ceiling effects.

In contrast, all items within the Workplace Harassment factor (Items 35–38) demonstrated elevated RI values (1.88–1.94), exceeding the recommended upper threshold. This pattern indicates that these items were frequently endorsed, potentially limiting response variability and reducing differentiation across respondents (see Table 7). These findings suggest that Workplace Harassment items may have limited discriminatory power in a general employed population, where exposure to harassment is relatively infrequent. Future studies should examine these items in larger and higher-risk occupational samples and consider additional sensitivity analyses, including item response theory or subgroup-based analyses.

Overall, the RI analysis supports the appropriateness of most MPWES items with respect to endorsement distribution and response variability. At the same time, the findings identify several items, particularly within the Social Support and Workplace Harassment dimensions, that may be susceptible to ceiling effects and therefore warrant further examination in subsequent stages of scale refinement and validation.

#### 5.3.11. Discrimination Index (DI)

The Discrimination Index (DI) was used to evaluate each item’s ability to distinguish between respondents with lower and higher levels of the underlying latent construct. DI values ranging from 0.20 to 0.80 were considered acceptable [18].

The results demonstrated that all MPWES items exhibited DI values within the recommended range (0.20–0.80), indicating acceptable discrimination across items and supporting their ability to adequately differentiate respondents by the level of the measured construct (see Table 8).

Overall, the observed DI range suggests that the items demonstrated satisfactory sensitivity to individual differences. No items demonstrated critically low or excessively elevated discrimination values indicating substantial psychometric concerns.

## 6. Discussion

The aim of this study was to develop and conduct an initial validation of the Multidimensional Psychosocial Work Environment Scale for Employed Persons (MPWES) by examining its content validity, face validity, factor structure, and key psychometric properties. Accordingly, the findings should be interpreted within the context of an initial, theory-driven validation process rather than as definitive evidence of a final measurement structure.

In line with the multidimensional logic of the MPWES, the discussion focuses not only on overall model adequacy, but also on the conceptual integration of the dimensions, the interpretation of comparatively weaker subscales, and the degree to which conceptually related factors remain theoretically and empirically distinguishable.

### 6.1. Content Validity and Expert Evaluation

The initial item pool of the MPWES demonstrated strong preliminary content validity, consistent with established principles of psychometric scale development, in which expert consensus during early validation stages is considered an important indicator of conceptual adequacy [15,22]. Like previous studies on the development of employee well-being instruments, higher conceptual clarity and agreement were observed for work-related resource dimensions such as social support, autonomy, and professional development, which are often described as relatively universal and empirically stable across occupational contexts [23,24].

In contrast, dimensions associated with work-related risks and adverse experiences, including Work-related Psychosomatic Strain and Financial Safety, demonstrated greater variability in expert judgments, reflecting the contextual sensitivity and methodological complexity involved in operationalizing these constructs, as noted in previous research [25]. Importantly, expert feedback primarily focused on item wording and semantic precision rather than conceptual misalignment, consistent with best-practice recommendations emphasizing iterative refinement during the early stages of scale development [16,26].

The achievement of full expert agreement following item revision further suggests that the MPWES demonstrates a content validity profile comparable to that reported for internationally used psychosocial work environment and employee well-being instruments, thereby providing a useful methodological basis for subsequent psychometric validation.

### 6.2. Face Validity

The face validity findings indicate that the MPWES items were generally perceived by respondents as clear, understandable, and linguistically appropriate. This finding is consistent with methodological recommendations that emphasize that the successful operationalization of theoretical constructs into comprehensible item wording is an important prerequisite for obtaining reliable psychometric assessments in self-report instruments [22,26].

Comparable findings have been reported in the development and validation of other psychosocial work environment and employee well-being instruments. Validation studies of the Copenhagen Psychosocial Questionnaire (COPSOQ) and related scales similarly demonstrate that most items typically achieve high perceived clarity, while minor variability in interpretation across selected items is common in instruments intended for broad and heterogeneous occupational populations [2,24].

Research on work engagement and occupational well-being instruments further suggests that face validity primarily serves a linguistic and interpretative refinement function rather than indicating the need for structural modifications of the scale itself [27]. Consequently, the high face validity observed in the present study provides additional support for the interpretability and practical applicability of the MPWES within occupational settings.

### 6.3. Confirmatory Factor Analysis and Model Stability

The CFA findings should be interpreted as providing preliminary and partial support for the hypothesized MPWES measurement structure rather than evidence of optimal model fit. The incremental fit indices obtained in the present study (CFI = 0.85; TLI = 0.84) were below commonly recommended conventional thresholds, indicating limited-to-moderate model fit. Therefore, the present CFA results should be interpreted with caution in the context of an initial theory-driven validation study.

At the same time, the RMSEA and SRMR values approached the commonly accepted thresholds, suggesting a borderline-to-acceptable model fit. This mixed pattern of fit indices indicates that the proposed ten-factor structure is theoretically interpretable but not yet fully optimized in terms of psychometric fit. Similar patterns may occur during the early validation of complex multidimensional psychosocial and well-being instruments, particularly when models include multiple theoretically distinct but conceptually related domains [19].

Several factors may explain the suboptimal incremental fit indices. First, the MPWES model includes ten dimensions, which increases model complexity. Second, the instrument contains heterogeneous indicator types, including Likert-type and dichotomous items. Third, several dimensions are represented by only two or a small number of items, which may reduce the stability of parameter estimates. Fourth, localized psychometric concerns were identified, particularly weak performance on the Financial Safety dimension and a high correlation between Inclusion and Social Support.

Importantly, the sequential model refinement analyses demonstrated that structural modifications, such as item removal, removal of the Financial Safety factor, or combining Inclusion and Social Support into a broader Social Resources factor, did not result in meaningful improvements in global model fit or construct interpretability. Therefore, the original theory-driven model was retained as the most conceptually coherent and empirically interpretable preliminary specification.

This decision should not be interpreted as confirmation that the ten-factor model is final. Rather, the identified model limitations indicate areas requiring further refinement and independent validation. The CFA findings provide a useful methodological foundation for continued scale optimization, but future studies should re-examine the MPWES factor structure in larger and independent samples, including exploratory factor analysis, exploratory structural equation modeling, and cross-validation before the measurement model is considered fully established.

### 6.4. Factor Loadings and Construct Representation

The standardized factor-loading analysis indicated that most MPWES items adequately represented their respective latent constructs, supporting the instrument’s conceptual coherence. Particularly stable loadings were observed for resource-oriented dimensions such as Subjective Well-Being, Autonomy, and Professional Development. This pattern is consistent with prior research suggesting that job resources are generally more readily operationalized and tend to demonstrate greater empirical stability than economically or socially sensitive constructs [2,24].

Comparatively weaker loadings observed for selected Social Support and Financial Safety items are methodologically understandable and have been documented in previous employee well-being instruments. The literature suggests that lower factor loadings within context-dependent psychosocial constructs do not necessarily indicate item inadequacy but may instead reflect the multidimensional and situationally sensitive nature of the construct itself [19].

Overall, the observed factor-loading pattern supports the interpretation that the MPWES dimensions generally represent the constructs satisfactorily, while also identifying several items that require targeted refinement in future validation stages.

### 6.5. Discriminant Validity and Interfactor Relationships

The discriminant validity findings generally support adequate construct distinctiveness across most MPWES dimensions, as most inter-factor correlations fell within the weak to moderate range. However, the strongest association was observed between Inclusion and Social Support (r = 0.88), indicating a high degree of empirical overlap between these two social resource dimensions.

This finding should be interpreted cautiously. Although Inclusion and Social Support are theoretically distinguishable, their discriminant validity is only partially supported in the present initial validation study. Inclusion refers primarily to perceived belonging, participation, and involvement in organizational processes, whereas Social Support refers to emotional and instrumental assistance from colleagues and supervisors. Thus, the two constructs represent conceptually distinct but closely related aspects of the psychosocial work environment.

The elevated correlation between Inclusion and Social Support, together with AVE values below the recommended threshold for both constructs, suggests that respondents may partly perceive these dimensions as interconnected components of a broader social work environment. However, this association should not be interpreted as complete conceptual redundancy. Rather, it reflects the naturally interconnected nature of social resources at work.

To further examine this issue, an alternative CFA specification combining Inclusion and Social Support into a single Social Resources factor was evaluated during the sequential model refinement process. This alternative model did not produce a meaningful improvement in global model fit or construct interpretability compared with the original specification. Therefore, Inclusion and Social Support were retained as separate but closely related dimensions in the preliminary MPWES structure.

Comparable empirical overlap has been documented in other employee well-being and psychosocial work environment instruments, in which social resource dimensions are conceptualized as closely related yet theoretically distinct constructs [28,29]. The observed interrelationships among job demand- and risk-related factors, together with their negative correlations with resource-oriented constructs, were generally consistent with the theoretical assumptions of the Job Demands–Resources (JD–R) framework [23].

Overall, the correlation pattern supports the instrument’s theoretical coherence while also indicating that the discriminant boundary between Inclusion and Social Support warrants further evaluation. Future validation studies should examine this issue using additional approaches, such as the heterotrait–monotrait ratio (HTMT), exploratory structural equation modeling, and cross-validation in independent samples, to determine whether these dimensions should remain separate or be represented as a broader Social Resources construct in future versions of the MPWES.

### 6.6. Convergent Validity and Construct Reliability

The convergent validity and construct reliability indicators were acceptable for most MPWES dimensions, indicating generally stable relationships between observed indicators and their underlying latent constructs. Higher AVE values were observed for dimensions such as Work-related Psychosomatic Strain, Workplace Harassment, and Autonomy, which are often described in the literature as more concrete and experience-based constructs demonstrating clearer empirical representation and greater internal homogeneity [25,30].

In contrast, lower AVE values observed for the Social Support and Inclusion dimensions corresponded with their elevated inter-factor correlation, suggesting partial conceptual overlap. Similar patterns are frequently reported in the assessment of social and relational constructs, which inherently involve interconnected interpersonal processes and context-dependent experiences.

The Financial Safety dimension demonstrated comparatively weaker reliability despite acceptable AVE values. This finding may reflect the construct’s contextual and exposure-oriented nature, as well as structural limitations of short scales during early validation phases. Taken together, these patterns support the interpretation that several MPWES dimensions function as contextual psychosocial constructs for which classical convergent validity criteria may be comparatively less informative during early stages of scale development.

### 6.7. Internal Consistency Reliability: Cronbach’s Alpha and McDonald’s Omega

Higher Cronbach’s alpha and McDonald’s omega values observed for dimensions such as Subjective Well-Being, Inclusion, Social Support, and Work Intensity are consistent with findings reported in the validation of COPSOQ, UWES, and similar multidimensional occupational well-being instruments, where these constructs are characterized by relatively stable and empirically homogeneous item structures [2,28,30].

In contrast, lower alpha and omega values were observed for factors represented by a limited number of items, particularly Workplace Harassment and Financial Safety. Comparable findings have been reported in previous research, indicating that sensitive, infrequently occurring, or contextually variable aspects of occupational experience often exhibit lower internal consistency when assessed with short scales [25,31].

In such contexts, lower reliability coefficients are typically not interpreted as indicators of construct inadequacy but rather as structural consequences of scale length and measurement design. Importantly, the Cronbach’s alpha if item deleted analyses did not reveal meaningful improvements in internal consistency, suggesting that reduced alpha values were not primarily attributable to isolated item-level problems.

This interpretation aligns with the methodological literature, which indicates that constrained alpha values often reflect the scale’s structural properties rather than deficiencies in item quality [32]. The inclusion of McDonald’s omega additionally provided a more robust estimate of reliability, as omega is less sensitive to violations of the tau-equivalence assumption and is therefore recommended for multidimensional psychometric instruments [19,33].

### 6.8. Item-Level Performance: Response Index (RI) and Discrimination Index (DI)

The item-level analyses using the Response Index (RI) and Discrimination Index (DI) provided additional evidence of the psychometric adequacy of the MPWES items and complemented the latent-variable findings from CFA and reliability analyses.

Overall, the observed RI values suggest that most items showed balanced endorsement distributions and adequate use of the response scales. Within resource-oriented dimensions, most Likert-type items fell within the recommended RI range, indicating sufficient variability and the absence of pronounced floor effects.

However, several items within the Social Support dimension demonstrated elevated RI values, suggesting potential ceiling effects. Similar response patterns have been documented in psychosocial work environment instruments, where positively framed social resource items are frequently highly endorsed in employed populations, thereby reducing variability without necessarily compromising conceptual relevance [29,30].

Importantly, such ceiling tendencies are commonly interpreted as reflecting the normative nature of social support constructs rather than item inadequacy. During early validation stages, these response patterns are generally considered methodologically acceptable because the primary objective is adequate construct representation rather than optimization of score distribution.

For dichotomous indicators, RI values generally remained within recommended thresholds, supporting balanced endorsement levels and appropriate item functioning. Elevated RI values observed for Workplace Harassment items are methodologically consistent with prior occupational health findings, as low-frequency yet highly salient adverse workplace experiences often skew response distributions [34,35].

The Discrimination Index findings further demonstrated that all items adequately differentiated between respondents with lower and higher levels of the underlying constructs. No items demonstrated discrimination values that were critically low or excessively elevated, indicating substantial psychometric concerns. Comparable discrimination performance has been reported in established occupational well-being instruments, including COPSOQ and UWES, in which adequate item discrimination is prioritized over maximizing response variability during early validation phases [2].

## 7. Limitations of the Study

Several limitations of the present study should be acknowledged when interpreting the findings. First, the study employed a non-probability convenience sampling approach, which may limit the generalizability of the results beyond the included occupational sectors and organizational contexts. Although participants were recruited from multiple sectors to increase the heterogeneity in psychosocial work environments, the sample was restricted to employed adults in Latvia; therefore, the cross-cultural applicability of the MPWES remains to be established.

Second, the study used a cross-sectional design, which precludes drawing conclusions about temporal stability, causal relationships, or the predictive validity of the identified psychosocial work environment dimensions. Consequently, future longitudinal studies are necessary to evaluate the stability of the factor structure over time and to examine the instrument’s sensitivity to changes in psychosocial work conditions and employee well-being.

Third, all data were self-reported, which may increase the risk of common method bias, response tendencies, and subjective interpretation of workplace experiences. Although anonymity and voluntary participation were emphasized to reduce social desirability bias, the possibility of reporting bias cannot be entirely ruled out.

Fourth, the psychometric evaluation was conducted on a single sample of 213 participants, which is relatively modest given the complexity of the proposed measurement model, comprising 45 items and 10 latent factors. Although a post hoc power analysis indicated adequate power to evaluate global model fit, the sample size limits the precision and stability of parameter estimates, particularly for a multidimensional CFA model with several short subscales and heterogeneous indicator types. In addition, the MPWES was developed and initially validated within the same sample, and no independent validation sample was available. Therefore, the present findings should be interpreted as preliminary, and replication, cross-validation, and further psychometric testing in larger independent occupational samples are necessary before firm conclusions can be drawn regarding the stability of the MPWES factor structure.

Fifth, exploratory factor analysis (EFA) was not conducted before confirmatory factor analysis (CFA). Although the MPWES measurement structure was specified a priori based on established theoretical frameworks and a theory-driven domain–item mapping process, EFA is commonly recommended during the early development of new psychometric instruments to empirically explore the latent structure before confirmatory testing. Therefore, the absence of EFA limits the extent to which alternative factor solutions could be examined in the present study. Future research should evaluate the MPWES using EFA, exploratory structural equation modeling, and cross-validation in independent and larger occupational samples before the factor structure is considered fully established.

In addition, the CFA model demonstrated limited-to-moderate fit, as the incremental fit indices (CFI = 0.85; TLI = 0.84) were below commonly recommended thresholds. Although RMSEA and SRMR indicated a borderline-to-acceptable fit, the overall model fit should therefore be interpreted with caution. The suboptimal fit may be partly explained by the complexity of the ten-factor model, the inclusion of heterogeneous indicator types, the presence of short subscales, the weak psychometric performance of the Financial Safety dimension, and the high correlation between Inclusion and Social Support. Future studies should re-evaluate the MPWES measurement structure in larger and independent samples before the proposed ten-factor model is considered psychometrically established.

Moreover, several dimensions were represented by only two or three items, including Financial Safety, Autonomy, Work-related Psychosomatic Strain, Health Risks, and Professional Development. Short subscales may reduce the stability of reliability estimates and may provide limited content coverage of the underlying constructs. This issue is particularly relevant for dimensions that represent complex or context-sensitive psychosocial domains, such as Financial Safety and Work-related Psychosomatic Strain. Therefore, these domains should be considered preliminary and interpreted cautiously at this initial validation stage. Future studies should expand these subscales with additional theoretically grounded items, test alternative item formulations, and evaluate whether these dimensions are best represented as reflective, formative, or exposure-based measurement components.

Furthermore, discriminant validity concerns were identified between the Inclusion and Social Support dimensions, as reflected by their elevated inter-factor correlation and AVE values below the recommended threshold. Although the distinction between these constructs is theoretically meaningful within the Job Demands–Resources (JD–R) framework, the present findings indicate only partial empirical support for their discriminant validity. Future studies should further evaluate whether Inclusion and Social Support should be retained as separate dimensions or combined into a broader Social Resources factor. Additional analyses, including the heterotrait–monotrait ratio (HTMT), exploratory structural equation modeling, and cross-validation in independent samples, are recommended to clarify the discriminant boundaries between these constructs.

Another limitation is that criterion-related validity was not assessed in the present study. Although the MPWES was theoretically informed by established frameworks and instruments, such as the WHO-5 Well-Being Index, the Job Demands–Resources model, the OECD job quality framework, and COPSOQ, the study did not include parallel administration of external validated measures. Therefore, associations between MPWES dimensions and established instruments could not be examined. Future studies should evaluate criterion-related validity by correlating MPWES dimensions with validated measures of well-being, psychosocial work environment, job demands, job resources, burnout, work engagement, and occupational stress. Furthermore, the present study did not directly test whether the integrated MPWES framework offers practical or psychometric advantages over the combined use of existing validated instruments. Future studies should therefore compare the MPWES with established measures such as COPSOQ, QPSNordic, and WHO-5 to evaluate its incremental validity, practical usefulness, and psychometric efficiency. Such analyses would provide stronger evidence regarding the external validity and practical interpretability of the MPWES.

Finally, the present study focused on the initial validation of the MPWES and did not assess test–retest reliability, measurement invariance, or longitudinal predictive validity. These psychometric properties should be examined in future research before broader application of the instrument in occupational health monitoring and organizational assessment contexts.

Despite these limitations, the study provides important preliminary evidence supporting the conceptual adequacy and psychometric potential of the MPWES as a multidimensional instrument for assessing psychosocial work environment factors associated with employee well-being.

## 8. Implications

The Multidimensional Psychosocial Work Environment Scale for Employed Persons (MPWES) represents a preliminary multidimensional instrument designed to assess psychosocial resources, job demands, and workplace-related risks across occupational and organizational settings. Given the initial validation design of the present study, the practical implications of the MPWES should be interpreted cautiously.

The multidimensional structure of the MPWES may potentially support a more nuanced understanding of psychosocial work environment factors associated with employee subjective well-being compared with global or single-score well-being measures. However, the current findings do not yet provide sufficient evidence to support the use of the MPWES as a diagnostic instrument or as a stand-alone basis for organizational decision-making.

If supported by further validation, the separate assessment of resource-, demand-, and risk-related dimensions may help researchers and occupational health professionals identify psychosocial domains warranting closer examination. In this sense, the MPWES may contribute to future psychosocial work environment assessment and monitoring, but its applied use in workplace interventions requires additional empirical testing.

The potential use of the MPWES in organizational prevention strategies, intervention evaluation, workplace sustainability assessment, or ESG-related social monitoring should therefore be regarded only as a possible future application rather than as an established outcome of the present study. These applications require further empirical evaluation before any firm, practical, or policy-related conclusions can be drawn.

Longitudinal, cross-cultural, and applied validation studies are needed to determine whether the instrument can reliably support organizational monitoring, intervention planning, or evaluation of changes in psychosocial work environment conditions over time.

Overall, the present study provides preliminary evidence for the conceptual and psychometric potential of the MPWES, while further validation is required before broader practical implementation in occupational health management, organizational decision-making, or workplace intervention contexts.

## 9. Conclusions

This study presents the development and initial validation of the Multidimensional Psychosocial Work Environment Scale for Employed Persons (MPWES). The findings provide preliminary empirical support for the instrument’s content validity, face validity, theoretically specified factor structure, and initial psychometric properties.

The results support the conceptual relevance of assessing the psychosocial work environment as a multidimensional construct integrating work-related resources, job demands, psychosocial risks, and employees’ subjective well-being within a unified preliminary measurement framework.

However, the present findings should be interpreted cautiously. Several dimensions require further refinement, particularly those represented by few items or showing weaker psychometric performance. In addition, the proposed factor structure requires confirmation in larger and independent samples before the MPWES can be considered a fully established measurement instrument.

Overall, the MPWES represents a psychometrically promising preliminary instrument for assessing psychosocial work environment characteristics associated with employee well-being. Further longitudinal, cross-cultural, and applied validation studies are needed to examine its stability, external validity, practical usefulness, and potential application in occupational health and organizational assessment contexts.

## Figures and Tables

**Table 1:** Comparative Overview of Theoretical Frameworks and Their Relevance for MPWES Development.

Framework/Instrument	Primary Focus	Main Contribution	Relevance for MPWES Development
PERMA [5]	Psychological flourishing and positive functioning	Emphasizes positive psychological functioning, meaning, engagement, and accomplishment	Informed positive functioning and subjective well-being dimensions
WHO-5 [7]	Emotional subjective well-being	Provides a brief and validated assessment of emotional well-being	Informed subjective well-being dimension
JD-R Model [1]	Job demands and job resources	Provides a theoretical framework linking workplace demands and resources with employee health and motivation	Informed of job demands and job resources dimensions
OECD Job Quality Framework [8]	Job quality and work-related quality of life	Provides a multidimensional perspective on work quality, workplace conditions, and sustainability	Informed organizational and psychosocial work environment domains
COPSOQ [2]	Psychosocial work environment	Comprehensive assessment of psychosocial workplace conditions and risks	Served as a methodological and comparative reference for identifying psychosocial work environment domains and informing item content; not treated as a primary theoretical framework.

Note. The listed theoretical frameworks and instruments informed the theory-driven development of the MPWES dimensions and item pool. The instrument was developed as an integrated psychosocial work environment assessment tool intended to evaluate psychosocial risks, job resources, workplace conditions, and dimensions associated with employee subjective well-being across occupational settings.

**Table 2:** Dimensional comparison of the MPWES with established psychosocial work environment and well-being instruments.

Instrument	Primary Focus	Work Related Resources	Job Demands	Psychosocial or Occupational Risks	Subjective Well-Being	Integrated Interpretation of Work Environment and Well-Being
COPSOQ-III	Comprehensive psychosocial work environment assessment	Yes	Yes	Yes	Partly	It provides a broad assessment of psychosocial working conditions but is not specifically organized as an integrated framework that combines selected work-environment dimensions with an outcome-related subjective well-being dimension.
QPSNordic	Psychological and social factors at work	Yes	Yes	Yes	Limited	Provides a structured assessment of psychological and social work factors, with less emphasis on subjective well-being as an integrated dimension within the same framework.
WHO-5 Well-Being Index	Emotional subjective well-being	No	No	No	Yes	Provides a brief assessment of subjective well-being, but does not assess workplace resources, job demands, or psychosocial risks.
MPWES	Integrated psychosocial work environment and subjective well-being assessment	Yes	Yes	Yes	Yes	Integrates selected workplace resources, job demands, psychosocial and occupational risk-related dimensions, and subjective well-being within one preliminary multidimensional assessment framework.

Note: The MPWES is not intended to replace established instruments such as COPSOQ-III, QPSNordic, or WHO-5. Its proposed added value lies in the theory-driven integration of selected psychosocial work environment factors and an outcome-related dimension of subjective well-being within a single preliminary assessment framework. However, whether this integrated approach offers practical or psychometric advantages over the combined use of existing validated instruments remains an open question and warrants examination in future studies.

**Table 3:** Theory-Driven Mapping of MPWES Domains and Illustrative Item Examples.

Theoretical Domain	Conceptual Definition	Illustrative Item Example (Non-Final Wording)	Theoretical Framework
Subjective well-being	Emotional well-being, positive psychological functioning, and overall subjective well-being in occupational settings	“I have felt cheerful and in good spirits.”	WHO-5
Inclusion	Sense of belonging, involvement in workplace processes, and perceived organizational inclusion	“How often do you feel involved in improving work processes?”	OECD/JD-R
Social support	Perceived emotional and instrumental support from colleagues and supervisors	“How often do you feel supported by your peers or colleagues?”	JD-R
Workplace harassment	Exposure to bullying, discrimination, harassment, or other negative workplace behaviors	“In the past 12 months, have you experienced workplace bullying or social exclusion?”	JD-R
Work intensity	Workload, time pressure, pace of work, and intensive job demand	“How often does your work involve working to tight deadlines?”	JD-R
Work-related psychosomatic strain	Psychological and physical strain symptoms associated with work-related stress exposure	“In the past 12 months, have you experienced mental health problems?”	JD-R
Professional development	Opportunities for learning, skills development, and career advancement	“In the past 12 months, have you received training aimed at improving your future work prospects?”	OECD/JD-R
Health risks	Exposure to physical, ergonomic, and occupational health risks	“How often are you exposed to noise at work?”	OECD
Financial safety	Perceived financial stability, income adequacy, and economic security	“I feel financially secure about my situation over the next six months.”	OECD
Autonomy	Degree of control over work tasks, decision-making, and work organization	“How often can you choose or change the methods of work?”	JD-R

Note. Illustrative items were conceptually informed by existing validated occupational health and psychosocial work environment instruments and are presented for demonstrative purposes only. Final item wording and psychometric evaluation were established during subsequent stages of MPWES development and initial validation.

**Table 4:** Response Formats and Preliminary Interpretation of MPWES Dimensions.

Dimension	Response Format	Preliminary Interpretation
Subjective well-being	Six-point temporal frequency scale	Reflects comparatively lower, moderate, or higher levels of emotional well-being and positive psychological functioning
Inclusion	Six-point temporal frequency scale	Reflects perceived levels of organizational inclusion, participation, and sense of belonging
Social support	Six-point Likert-type agreement scale	Reflects perceived emotional and instrumental workplace support
Workplace harassment	Dichotomous (Yes/No)	Indicates the presence or absence of workplace harassment exposure
Work intensity	Six-point temporal frequency scale	Reflects perceived workload, time pressure, and intensive work demands
Work-related psychosomatic strain	Dichotomous (Yes/No)	Indicates the presence or absence of work-related psychological or physical strain symptoms
Professional development	Dichotomous (Yes/No)	Indicates the presence or absence of access to professional development opportunities
Health risks	Six-point temporal frequency scale	Reflects perceived exposure to occupational and workplace-related health risks
Financial safety	Six-point Likert-type agreement scale	Reflects perceived financial stability and economic security
Autonomy	Six-point temporal frequency scale	Reflects perceived levels of control, decision latitude, and task-related autonomy

Note. The proposed interpretive categories are preliminary and intended solely to facilitate descriptive interpretation during the initial validation stage. The MPWES is not intended for diagnostic classification, and definitive threshold values require further empirical validation with larger, more diverse occupational samples.

**Table 5:** Item-Level and Scale-Level Content Validity Indices Following the First Round of Expert Evaluation.

MPWES Domains	Item No.	Experts Rating Item as Relevant (≥3)	Experts Rating Item not Relevant (≤2)	I-CVI (Round 1)	S-CVI/Ave (Round 1)	P_c_	κ*
Subjective well-being	**6**	**4**	**2**	**0.67**	0.95		
	7	6	0	1.00		0.016	1.00
	15	6	0	1.00		0.016	1.00
	**22**	**5**	**1**	**0.83**		0.094	0.81
	41	6	0	1.00		0.016	1.00
	42	6	0	1.00		0.016	1.00
	43	6	0	1.00		0.016	1.00
	44	6	0	1.00		0.016	1.00
	45	6	0	1.00		0.016	1.00
Inclusion	9	6	0	1.00	0.96	0.016	1.00
	13	6	0	1.00		0.016	1.00
	14	6	0	1.00		0.016	1.00
	**16**	**5**	**1**	**0.83**		0.094	0.81
	17	6	0	1.00		0.016	1.00
	**18**	**5**	**1**	**0.83**		0.094	0.81
	20	6	0	1.00		0.016	1.00
	21	6	0	1.00		0.016	1.00
	31	6	0	1.00		0.016	1.00
Social support	1	6	0	1.00	0.96	0.016	1.00
	2	6	0	1.00		0.016	1.00
	3	6	0	1.00		0.016	1.00
	4	6	0	1.00		0.016	1.00
	5	6	0	1.00		0.016	1.00
	**8**	**5**	**1**	**0.83**		0.094	0.81
	**12**	**5**	**1**	**0.83**		0.094	0.81
	19	6	0	1.00		0.016	1.00
Workplace harassment	**35**	**4**	**2**	**0.67**	0.79	0.234	0.57
	**36**	**5**	**1**	**0.83**		0.094	0.81
	37	6	0	1.00		0.016	1.00
	**38**	**5**	**1**	**0.83**		0.094	0.81
Work intensity	25	6	0	1.00	0.92	0.016	1.00
	**26**	**5**	**1**	**0.83**		0.094	0.81
	**29**	**5**	**1**	**0.83**		0.094	0.81
	30	6	0	1.00		0.016	1.00
Work-related psychosomatic strain	**39**	**4**	**2**	**0.67**	0.67	0.234	0.57
	**40**	**4**	**2**	**0.67**		0.234	0.57
Professional development	32	6	0	1.00	1.00	0.016	1.00
	33	6	0	1.00		0.016	1.00
	34	6	0	1.00		0.016	1.00
Health risks	23	6	0	1.00	0.84	0.016	1.00
	**24**	**4**	**2**	**0.67**		0.234	0.57
Financial safety	**10**	**4**	**2**	**0.67**	0.67	0.234	0.57
	**11**	**4**	**2**	**0.67**		0.234	0.57
Autonomy	**27**	**5**	**1**	**0.83**	0.92	0.094	0.81
	28	6	0	1.00		0.016	1.00

Note. I-CVI denotes the Item-level Content Validity Index, and S-CVI/Ave represents the Scale-level Content Validity Index calculated using the average method. Pc indicates the probability of chance agreement, and κ* refers to the modified kappa coefficient. Items identified for revision based on expert feedback were subsequently revised and resubmitted for reassessment in Round 2. Bold values indicate items that required revision after the first round of expert evaluation and were subsequently reassessed in Round 2. Detailed expert-level CVI values for the full instrument are presented in Supplementary Material Table S2.

**Table 6:** Item-Level Face Validity Indices Based on Respondent Evaluation (*n* = 5).

Item No.	R1	R2	R3	R4	R5	SUM	I-FVI
1	1	0	1	1	1	4	0.80
2	1	1	1	1	0	4	0.80
3	1	1	1	1	1	5	1.00
4	1	1	1	1	0	4	0.80
5	1	1	1	1	1	5	1.00
6	1	0	1	1	1	4	0.80
7	1	1	1	1	1	5	1.00
8	1	1	1	1	1	5	1.00
9	1	1	1	1	1	5	1.00
10	1	0	1	1	1	4	0.80
11	1	1	1	1	1	5	1.00
12	1	1	1	1	1	5	1.00
13	1	1	1	1	1	5	1.00
14	1	1	0	1	1	4	0.80
15	1	1	1	1	1	5	1.00
16	1	1	1	1	1	5	1.00
17	1	1	1	1	1	5	1.00
18	1	1	1	1	1	5	1.00
19	1	1	1	1	1	5	1.00
20	1	1	1	1	1	5	1.00
21	0	1	1	1	1	4	0.80
22	1	0	1	1	1	4	0.80
23	1	1	1	1	1	5	1.00
24	1	1	1	1	1	5	1.00
25	1	1	1	1	1	5	1.00
26	1	1	1	1	1	5	1.00
27	1	1	1	1	0	4	0.80
28	1	0	1	1	1	4	0.80
29	1	1	1	1	1	5	1.00
30	1	1	1	1	1	5	1.00
31	1	1	1	1	1	5	1.00
32	1	1	1	1	1	5	1.00
33	0	1	1	1	1	4	0.80
34	1	1	1	1	1	5	1.00
35	1	1	1	1	1	5	1.00
36	1	1	1	1	1	5	1.00
37	1	1	1	1	1	5	1.00
38	1	1	1	1	1	5	1.00
39	1	1	1	1	1	5	1.00
40	1	1	0	1	1	4	0.80
41	1	1	1	1	1	5	1.00
42	1	1	1	1	1	5	1.00
43	1	1	1	1	1	5	1.00
44	1	1	1	1	1	5	1.00
45	1	1	1	1	1	5	1.00
FVI	0.95	0.91	0.96	0.98	0.95	-	0.95

Note. I-FVI = Item-level Face Validity Index; *n* = 5 respondents. I-FVI values were calculated as the proportion of respondents rating each item as clear and comprehensible.

**Table 7:** Standardized Factor Loadings for the MPWES CFA Measurement Model.

Factor	Number of Items	Range of Standardized Loadings (β)	Interpretation (Items < 0.50 Explicitly Indicated)
Subjective well-being	9	0.63–0.87	All items demonstrated acceptable loadings
Inclusion	8	0.53–0.79	All items demonstrated acceptable loadings
Social support	8	0.48–0.71	One item (5, β = 0.48) falls below the recommended threshold, indicating a comparatively weaker association with the latent factor
Workplace harassment	4	0.86–0.88	All items demonstrated acceptable loadings
Work intensity	4	0.73–0.97	All items demonstrated acceptable loadings
Work-related psychosomatic strain	2	0.77–0.88	All items demonstrated acceptable loadings
Professional development	3	0.96–0.96	All items demonstrated strong loadings
Health risks	2	0.78–0.95	All items demonstrated acceptable loadings
Financial safety	2	0.45–1.11	One item (11, β = 0.45) falls below the threshold; the second item (10, β > 1.00) indicates potential estimation instability
Autonomy	2	0.79–0.88	All items demonstrated acceptable loadings

Note. β denotes standardized factor loadings obtained from confirmatory factor analysis (CFA). Loadings ≥ 0.50 were considered acceptable. Items with standardized loadings below this threshold are explicitly indicated in the Interpretation column. Loadings exceeding 1.00 may indicate instability in estimation or specification.

**Table 8:** Item-Level Reliability and Diagnostic Indicators of the MPWEP Scales.

Item	RI	DI	Cronbach’s α if Item Deleted	Cronbach’s α Factor	McDonald’s Omega	Number of Items
Subjective well-being						
6	4.40	0.53	0.90	0.91	0.91	9
7	4.37	0.63	0.89			
15	4.43	0.68	0.89			
22	4.23	0.88	0.89			
41	4.13	0.70	0.89			
42	4.01	0.71	0.89			
43	4.06	0.76	0.88			
44	3.79	0.73	0.89			
45	4.18	0.70	0.89			
Inclusion						
9	4.20	0.53	0.83	0.85	0.85	9
13	4.84	0.51	0.84			
14	4.69	0.51	0.84			
16	4.11	0.67	0.82			
17	4.87	0.56	0.83			
18	4.28	0.51	0.84			
20	3.24	0.49	0.84			
21	3.64	0.65	0.82			
31	4.43	0.64	0.82			
Social support						
1	4.94	0.51	0.79	0.81	0.81	8
2	4.06	0.57	0.78			
3	5.10	0.51	0.79			
4	4.89	0.63	0.77			
5	5.20	0.42	0.80			
8	5.04	0.52	0.79			
12	4.88	0.57	0.78			
19	4.90	0.48	0.79			
Workplace harassment						
35	1.94 *	0.50	0.58	0.67	0.66	4
36	1.88 *	0.53	0.55			
37	1.91 *	0.41	0.64			
38	1.94 *	0.40	0.64			
Work intensity						
25	3.04	0.58	0.75	0.80	0.77	4
26	3.65	0.57	0.76			
29	3.74	0.62	0.62			
30	3.85	0.64	0.64			
Work-related Psychosomatic Strain						
39	1.75 *	0.43	**	0.60	**	2
40	1.57 *	0.43	**			
Professional development						
32	1.34 *	0.50	0.80	0.78	0.79	3
33	1.38 *	0.65	0.65			
34	1.29 *	0.68	0.81			
Health risks						
23	2.56	0.67	**	0.80	**	2
24	2.80	0.67	**			
Financial safety						
10	2.06	0.32	**	0.49	**	2
11	2.40	0.32	**			
Autonomy						
27	3.69	0.65	**	0.79	**	2
28	4.00	0.65	**			

Note. RI = Response Index, representing the average level of item endorsement. For items measured on a 6-point Likert scale, acceptable RI values range from 2 to 5. For dichotomous items * (scored 1–2), the acceptable RI range is 1.20–1.80. DI = Discrimination Index; acceptable values range from 0.20 to 0.80 [18]. ** McDonald’s ω and Cronbach’s α if item deleted could not be calculated for factors consisting of fewer than three items, as reliability estimates are considered unstable under such conditions.

## Data Availability

The datasets generated and/or analysed during the current study are not publicly available due to ethical restrictions and the risk of identifying individual participants but are available from the corresponding author on reasonable request.

## Supplementary Materials

The following supporting information can be downloaded at: https://www.mdpi.com/article/10.3390/ijerph23070854/s1, File S1: Multidimensional Scale of Psychosocial Well-Being for Employed Persons (MPSWEP); File S2: Psihosociālās labbūtības daudzdimensionālā skala nodarbinātajām personām (PLDS-NP); Table S1: Expert ratings (4-point relevance scale)—Round 1; Table S2: Expert ratings (dichotomized relevance scale)—Round 1; Table S3: Item revisions following Round 1 expert content validity assessment.
