# Supplementary material for: Development and Initial Validation of the Multidimensional Psychosocial Work Environment Scale for Employed Persons (MPWES)
Source: Int J Environ Res Public Health. 2026 Jun 30;23(7):854. doi: 10.3390/ijerph23070854 (PMC13409794; doi:10.3390/ijerph23070854)
Supplement: Supplementary file 1 [file ijerph-23-00854-s001.zip › Supplementary_3.pdf]

Table S1. Expert ratings (4-point relevance scale) - Round 1.

**Item No.**	Ex.1.	Ex.2.	Ex.3.	Ex.4.	Ex.5.	Ex.6.
1.	4	4	4	4	4	4
2.	4	4	4	4	4	4
3.	4	4	4	4	4	4
4.	4	4	3	4	4	4
5.	4	4	4	4	4	4
6	4	4	2	4	4	2
7.	4	4	4	4	4	4
8.	4	2	4	4	4	4
9.	4	4	4	4	4	4
10.	2	4	1	4	4	4
11.	2	3	2	4	4	4
12.	4	4	4	2	4	4
13.	4	4	4	4	4	4
14.	4	4	4	4	4	2
15.	4	4	4	4	4	4
16.	4	2	4	4	4	4
17.	4	4	4	4	4	4
18.	4	4	2	4	4	4
19.	4	4	4	4	4	4
20.	4	4	4	4	4	4
21.	4	4	4	4	4	4
22.	4	4	4	4	1	4
23.	4	4	4	4	4	1
24.	2	4	2	4	4	4
25.	4	4	4	4	4	4
26.	2	4	4	4	4	4
27.	2	4	4	4	4	4
28.	4	4	4	4	4	4
29.	4	4	4	2	4	4
30.	4	4	4	4	4	4
31.	4	4	4	4	4	4
32.	4	4	4	4	4	4
33.	4	4	4	4	4	4
34.	4	3	3	4	4	3
35.	4	4	2	4	2	4
36.	4	3	4	1	3	4
37.	4	3	3	4	4	3
38.	4	3	2	3	3	4
39.	4	2	3	4	1	3
40.	4	1	3	4	1	3
41.	4	4	4	4	4	4
42	4	4	4	4	4	4
43.	4	4	4	4	4	4
44.	4	3	4	3	4	4
45.	4	4	4	4	4	4

Table S2. Expert ratings (dichotomized relevance scale) - Round 1.

Item No.	Ex.1	Ex.2	Ex.3	Ex.4	Ex.5	Ex.6	SUM	**CVI**	**Decision**
1	1	1	1	1	1	1	6	1.00	Retain
2	1	1	1	1	1	1	6	1.00	Retain
3	1	1	1	1	1	1	6	1.00	Retain
4	1	1	1	1	1	1	6	1.00	Retain
5	1	1	1	1	1	1	6	1.00	Retain
6	1	1	0	1	1	0	4	0.67	Revise and retain
7	1	1	1	1	1	1	6	1.00	Retain
8	1	0	1	1	1	1	5	0.83	Revise and retain
9	1	1	1	1	1	1	6	1.00	Retain
10	0	1	0	1	1	1	4	0.67	Revise / Consider removal
11	0	1	0	1	1	1	4	0.67	Revise and retain
12	1	1	1	0	1	1	5	0.83	Revise and retain
13	1	1	1	1	1	1	6	1.00	Retain
14	1	1	1	1	1	1	6	1.00	Retain
15	1	1	1	1	1	1	6	1.00	Retain
16	1	0	1	1	1	1	5	0.83	Revise and retain
17	1	1	1	1	1	1	6	1.00	Retain
18	1	1	0	1	1	1	5	0.83	Revise and retain
19	1	1	1	1	1	1	6	1.00	Retain
20	1	1	1	1	1	1	6	1.00	Retain
21	1	1	1	1	1	1	6	1.00	Retain
22	1	1	1	1	1	0	5	0.83	Revise and retain
23	1	1	1	1	1	1	6	1.00	Retain
24	0	1	0	1	1	1	4	0.67	Revise and retain
25	1	1	1	1	1	1	6	1.00	Retain
26	0	1	1	1	1	1	5	0.83	Revise and retain
27	0	1	1	1	1	1	5	0.83	Revise and retain
28	1	1	1	1	1	1	6	1.00	Retain
29	1	1	1	0	1	1	5	0.83	Revise and retain
30	1	1	1	1	1	1	6	1.00	Retain
31	1	1	1	1	1	1	6	1.00	Retain
32	1	1	1	1	1	1	6	1.00	Retain
33	1	1	1	1	1	1	6	1.00	Retain
34	1	1	1	1	1	1	6	1.00	Retain
35	1	1	0	1	0	1	4	0.67	Revise / Consider removal
36	1	1	1	0	1	1	5	0.83	Revise and retain
37	1	1	1	1	1	1	6	1.00	Retain
38	1	1	0	1	1	1	5	0.83	Revise and retain
39	1	0	1	1	0	1	4	0.67	Revise / Consider removal
40	1	0	1	1	0	1	4	0.67	Revise / Consider removal
41	1	1	1	1	1	1	6	1.00	Retain
42	1	1	1	1	1	1	6	1.00	Retain
43	1	1	1	1	1	1	6	1.00	Retain
44	1	1	1	1	1	1	6	1.00	Retain
45	1	1	1	1	1	1	6	1.00	Retain
**CVI**	0.89	0.89	0.84	0.91	0.93	0.96	-	0.90	

Note. Items with an I-CVI of 1.00 were retained without modification. Items with an I-CVI of 0.83 were revised for clarity and retained, whereas items with an I-CVI of 0.67 were subject to substantial revision and further evaluation.

Table S3. Item revisions following Round 1 expert content validity assessment.

Item No.	Domain	Round 1 I-CVI	Main expert concern	Type of revision made	Round 2 I-CVI
**Item 6**	Subjective well-being	0.67	Item wording required clarification and stronger alignment with the subjective well-being domain.	Wording was clarified and conceptual alignment with emotional well-being and positive functioning was strengthened.	1.00
**Item 22**	Subjective well-being	0.83	Minor clarification was needed to improve interpretability.	Wording was refined to improve clarity while preserving the original theoretical meaning.	1.00
**Item 16**	Inclusion	0.83	Item wording required minor clarification regarding organizational involvement.	Wording was refined to better reflect participation and involvement in organizational processes.	1.00
**Item 18**	Inclusion	0.83	Minor ambiguity was identified in the interpretation of inclusion-related content.	Wording was clarified to strengthen alignment with the inclusion domain.	1.00
**Item 8**	Social support	0.83	Item wording required minor clarification regarding perceived support.	Wording was refined to improve interpretability of emotional or instrumental workplace support.	1.00
**Item 12**	Social support	0.83	Minor wording ambiguity was identified.	Wording was clarified to better reflect perceived support from colleagues or supervisors.	1.00
**Item 35**	Workplace harassment	0.67	Item wording was potentially ambiguous and required clearer reference to workplace harassment exposure.	Ambiguous wording was reduced and the item was revised to better reflect workplace harassment as a psychosocial risk.	1.00
**Item 36**	Workplace harassment	0.83	Minor clarification was needed regarding the type of negative workplace experience.	Wording was refined to improve clarity and consistency with the workplace harassment domain.	1.00
**Item 38**	Workplace harassment	0.83	Minor ambiguity was identified in the interpretation of workplace harassment-related content.	Wording was clarified to strengthen conceptual alignment with psychosocial risk exposure.	1.00
**Item 26**	Work intensity	0.83	Item wording required minor clarification regarding workload or time pressure.	Wording was refined to better reflect intensive work demands.	1.00
**Item 29**	Work intensity	0.83	Minor wording ambiguity was identified in relation to work intensity.	Wording was clarified to improve conceptual precision and interpretability.	1.00
**Item 39**	Work-related psychosomatic	0.67	The conceptual link between the item and work-related strain	Wording was revised to better reflect strain symptoms associated	1.00

	strain		required clarification.	with work-related stress exposure.	
**Item 40**	Work-related psychosomatic strain	0.67	Item wording required linguistic simplification and clearer interpretation.	Wording was simplified and interpretability was improved while preserving the original theoretical meaning.	1.00
**Item 24**	Health risks	0.67	Occupational health-risk content required clearer wording.	Wording was clarified to better reflect exposure to health-related occupational risks.	1.00
**Item 10**	Financial safety	0.67	The financial safety construct required clearer formulation.	Wording was revised to improve conceptual clarity and alignment with perceived financial stability.	1.00
**Item 11**	Financial safety	0.67	The item required stronger alignment with the financial safety domain.	Domain alignment was strengthened and wording was clarified to better reflect perceived economic security.	1.00
**Item 27**	Autonomy	0.83	Minor clarification was needed regarding decision latitude or control over work.	Wording was refined to improve alignment with the autonomy domain.	1.00

Note. I-CVI = Item-level Content Validity Index. Items listed in this table were identified as requiring revision after Round 1 expert assessment because they showed either comparatively lower or non-unanimous expert agreement. Revisions were based on experts' written comments and item-level relevance ratings. The revised items were re-evaluated by the same expert panel in Round 2, using the same four-point relevance scale and evaluation criteria.
